# Depressive symptoms precede and drive problematic smartphone use in Chinese medical students: a longitudinal network analysis

**DOI:** 10.3389/fpsyg.2026.1808905

**Published:** 2026-05-08

**Authors:** Wenhao Lv, Junjun Huang, Mengyuan Yang, Yajuan Zhang, Jun Wang, Hongliang Lu, Xia Zhu

**Affiliations:** Department of Military Medical Psychology, Air Force Medical University, Xi'an, Shaanxi, China

**Keywords:** cross-lagged panel network, depression, medical graduate students, network comparison, problematic smartphone use

## Abstract

**Background:**

Depression and problematic smartphone use (PSU) frequently co-occur in Chinese medical students and are increasingly attracting significant attention. Although the link between PSU and depression is well recognized, patterns of longitudinal dynamic associations at the symptom level remain poorly understood, particularly in the context of Chinese medical students.

**Methods:**

A total of 584 medical graduate students (mean age = 30.47 years, SD = 3.57; 65.2% males) completed the Smartphone Addiction Scale-Short Version and the 9-item Patient Health Questionnaire at two time points separated by 6 months. This study examined contemporaneous networks and cross-lagged panel network (CLPN) for both waves of data.

**Results:**

Network comparison test revealed that the two contemporaneous networks including PSU and depressive symptoms were consistent in global strength and network structure, with PHQ7 (“concentration difficulties”) and PHQ4 (“fatigue”) identified as bridging symptoms in both networks. The CLPN results indicated that the comorbidity was primarily driven by depressive symptoms at T1 triggering the development of PSU symptoms at T2. Among these, PHQ7 and PHQ4 exerted the greatest influence on other symptoms within the network and served as critical factors in activating the progression of PSU symptoms at T2. PSU4 (“can't stand not having a phone”) and PSU7 (“never give up using a phone”) were the most susceptible subsequent symptoms.

**Conclusions:**

Our findings provide empirical evidence supporting the comorbidity between PSU and depression, while elucidating their directional relationships at the symptom level. Crucially, we have identified antecedent bridge symptoms as effective intervention targets for simultaneously mitigating the development of co-occurring disorders.

## 1 Introduction

In the digital age, smartphones have become an indispensable part of daily life, offering unparalleled convenience and connectivity (Zhong et al., 2022). However, the pervasive use of smartphones has also given rise to problematic smartphone use (PSU), characterized by excessive and compulsive behaviors that interfere with daily functioning (Lin et al., 2017). PSU is widely conceptualized as a behavioral addiction and comprises four core components: compulsive behaviors; tolerance, withdrawal and functional impairment (Lin et al., 2014). PSU has been reported across diverse populations, with prevalence rates varying significantly depending on demographic factors such as age, occupation, and cultural context (Al-Mohaimeed et al., 2022; Lopez-Fernandez et al., 2018; Sanchez-Fernandez and Borda-Mas, 2023). Among graduate students, particularly those in high-stress environments like medical education, PSU has emerged as a growing mental health and productivity concern (Chen et al., 2017; Choujaa et al., 2023). Factors such as academic pressure, social isolation, and the need for constant connectivity contribute to the heightened risk of PSU in this population (Hao et al., 2022). The consequences of PSU are far-reaching, encompassing impaired academic performance, disrupted sleep patterns, and diminished mental well-being (Mei et al., 2023; Montag et al., 2015; Peltz et al., 2023). Given the growing prevalence of PSU and its potential to exacerbate existing stressors, there is an urgent need to investigate its longitudinal relationships with mental health outcomes among medical graduate students.

Depression is a prevalent mental health issue among medical graduate students, with studies indicating alarmingly high rates in this population (Guo et al., 2021; Ossai et al., 2021). The rigorous demands of medical training, coupled with the pressure to excel academically and clinically, create a fertile ground for the development of depressive symptoms (Shao et al., 2020). The results of a meta-analysis showed that the global prevalence of depression among medical students was 28.0% and was higher among graduate medical students (Puthran et al., 2016). Another study reported that the average prevalence of depression among Chinese medical students was 32.74 %, which may be due to the more intense academic competition and employment pressure in China (Mao et al., 2019). Existing research has found that depressive symptoms are highly correlated with a variety of psychological problems, such as anxiety, loneliness, and impulsivity (Yang et al., 2023; Yucens and Uzer, 2018). The effects of depression may not be limited to the symptoms themselves, but are also associated with a range of adverse outcomes, including burnout, reduced occupational efficacy and even suicidal ideation (Dyrbye et al., 2010; Grace, 2018; Zhou et al., 2021). Based on previous research results, there is a significant correlation between depressive and PSU symptoms (Ay et al., 2024; Jia et al., 2024). PSU may serve as both a coping mechanism for and a contributor to depressive symptoms, creating a vicious cycle that perpetuates mental health deterioration. Despite the recognized link between PSU and depression, the longitudinal dynamics of this relationship remain poorly understood, particularly in the context of Chinese medical graduate students. Understanding these dynamics is crucial for developing targeted interventions to mitigate the adverse effects of both PSU and depression.

The relationship between PSU and depressive symptoms has received increasing attention from researchers in recent years, and existing theoretical models such as the compensatory internet use theory and the interaction of person-affect-cognition-execution (I-PACE) model provide a framework for understanding the relationship between PSU and depression (Brand et al., 2019; Kardefelt-Winther, 2014). These models posit that individuals may turn to smartphone use as a maladaptive coping strategy to alleviate negative emotions, and spend more time on communication activities on mobile devices (Brand et al., 2016; Ye et al., 2025). However, reliance on smartphones will not guarantee that it will alleviate problems or solve longstanding issues. On the contrary, it may exacerbate psychological conditions or even drag users into unregulated and problematic smartphone use (Kim et al., 2015; Smetaniuk, 2014). In addition, a systematic review found that depression severity was consistently associated with problematic smartphone use and showed at least a moderate effect (Elhai et al., 2017). However, most studies to date have relied on cross-sectional designs, which limit inferences about causal or temporal relationships (Andrade et al., 2020; Ay et al., 2024; Chen et al., 2024). Although PSU is similar to generalized internet addiction, focusing on different devices and their patterns of behavioral use has important implications for the study of addictive behaviors and their harms (Lin et al., 2017). In summary, there is a paucity of longitudinal studies on the relationship between PSU and depression, and existing studies often fail to explain the complex dynamic interactions between PSU and depression. This gap underscores the need for longitudinal research that captures the evolving nature of PSU and depression.

Network analysis has emerged as a powerful tool for examining the intricate relationships between psychological constructs in recent years (Borsboom and Cramer, 2013). In contrast to traditional approaches that focus on latent variables, network analysis conceptualizes mental health problems as systems of interacting symptoms, with edges in the network representing associations between two nodes, while considering other variables in the network (Borsboom and Cramer, 2013; Epskamp et al., 2012). Studies have identified positive associations within the network of PSU and depressive symptoms, however there is a relative lack of in-depth research on the longitudinal associations between the two mental disorders (Huang et al., 2021; Mullarkey et al., 2019). Moreover, this approach allows for the identification of central symptoms that play pivotal roles in maintaining the network, as well as the detection of symptom clusters or communities (Beard et al., 2016). Centrality metrics provide insights into the relative importance of individual symptoms within the network, and the bridge centrality indicator, as an indicator for assessing the connectivity between different network associations, provides an important understanding of the interaction and dissemination relationships between different networks (Beard et al., 2016; Jones et al., 2021). The application of network analysis in psychology offers new perspectives on the structure and dynamics of mental health disorders, and provides deeper insights and more precise intervention targets for clinical intervention and treatment of psychological disorders.

While cross-sectional network analysis has advanced our understanding of the static relationships between PSU and depression, it remains limited in its ability to capture dynamic associations between individual symptoms. Cross-lagged panel network (CLPN) analysis addresses this limitation by modeling the longitudinal interplay between symptoms across multiple time points (Kan et al., 2025). In CLPN, nodes represent symptoms at different time points, and edges represent the temporal relationships between these symptoms (Wang et al., 2025; Ye et al., 2025). In the CLPN model, the autoregressive effects of the nodes and the cross-lagged effects between different nodes are observed based on the directed network structure graph (Wysocki et al., 2022). Furthermore, it provides out-expected influence (out-EI) and in-expected influence (in-EI) metrics to locate the sensitivity of nodes in networks across time, offering insights into potential intervention targets (Wysocki et al., 2022; Ye et al., 2025). This method also allows for the examination of reciprocal relationships, providing a more comprehensive understanding of how PSU and depression influence each other longitudinally. By employing CLPN, researchers can uncover the directional pathways through which PSU and depression interact, offering valuable insights for both theoretical development and clinical practice.

Given the limitations of existing research and the potential of CLPN, this study aims to investigate the longitudinal relationships between PSU and depression among Chinese medical graduate students using cross-sectional and cross-lagged panel network analysis. Specifically, the study has three objectives: (1) to determine whether there are changes in the network structure of PSU and depression comorbidity and in central symptoms across different time points, (2) to identify central and sensitive symptoms that drive the PSU and depression comorbidity networks over time, and (3) to examine whether the mechanisms underlying the pathogenesis of comorbidity can be explained by the predictive effect of prior depression on subsequent PSU, or vice versa. By addressing these objectives, this study seeks to contribute to the growing body of literature on PSU and depression, offering new insights into their longitudinal dynamics in a high-stress educational context. The findings have important theoretical implications for understanding the mechanisms underlying PSU and depression, while also offering practical implications for developing targeted mental health interventions. Ultimately, this study aims to provide strategies to mitigate the adverse effects of PSU and depression and to enhance the mental health and professional efficacy of the future graduate medical student population.

## 2 Method

### 2.1 Participants and procedure

This longitudinal study recruited graduate students through purposeful sampling from two medical universities in Xi'an, China. Eligible participants received detailed information regarding research objectives, duration, and confidentiality protocols prior to enrollment. Data collection was administered via WeChat, which is the predominant social media platform in China and is used almost universally by the university population. All participants voluntarily completed the baseline assessment at Time 1 (T1) in May 2023. To ensure data quality, we implemented an attention-check item and excluded responses with verification failures or implausibly short completion times. From 1,024 distributed surveys, 964 valid responses were retained (94.14%). Based on the existing research, we have chosen a time interval of 6 months (which is equivalent to one academic semester) (Hu et al., 2025). Through unique student identifier matching, 584 participants with complete longitudinal data were included in final analyses.

### 2.2 Measures

#### 2.2.1 Depressive symptoms

Depressive symptoms were assessed using the 9-item Patient Health Questionnaire (PHQ-9) (Diez-Quevedo et al., 2001). Each item evaluates the frequency of specific emotional experiences during the preceding 2 weeks, including core depressive symptoms such as “loss of interest or pleasure” and “feeling down, depressed, or hopeless.” Responses are recorded on a 4-point Likert scale ranging from 0 (“not at all”) to 3 (“nearly every day”), yielding a total score range of 0–27. Higher total scores indicate greater severity of depressive symptoms. The Chinese version of PHQ-9 has demonstrated robust psychometric properties in previous validation studies (Wang et al., 2014). In the current investigation, the scale exhibited excellent internal consistency, with Cronbach's α coefficients of 0.884 at T1 and 0.867 at T2.

#### 2.2.2 Problematic smartphone use

The severity of problematic smartphone use was measured using the Chinese version of the smartphone addiction scale-short version (SAS-SV) (Kwon et al., 2013), a unidimensional assessment tool. This 10-item scale evaluates core behavioral indicators of smartphone overuse, including statements such as “I cannot stand not having my smartphone” and “I constantly check my smartphone to avoid missing messages or news.” Responses are recorded on a 6-point Likert scale ranging from 1 (“strongly disagree”) to 6 (“strongly agree”), with total scores ranging from 10 to 60. The Chinese adaptation of SAS-SV has undergone rigorous cultural adaptation and validation processes within Chinese university populations, demonstrating satisfactory psychometric properties (Zhao et al., 2022). Notable modifications from the original version include linguistic refinements to enhance item comprehensibility among native Mandarin speakers. Reliability analyses revealed excellent internal consistency, with Cronbach's α coefficients of 0.902 at T1 and 0.928 at T2.

### 2.3 Statistical analysis

#### 2.3.1 Contemporaneous network analysis

First, we use the function “goldbricker” from the R package “networktools” to assess the potential redundancy of nodes (Jones, 2024); node pairs which have less than the 20% of significantly different correlations will be considered “bad pairs” (Everaert and Joormann, 2019; Rodriguez et al., 2022). Data analyses were conducted using R software (version 4.4.1) with specialized packages. The graphical least absolute shrinkage and selection operator with extended Bayesian information criterion (EBICglasso) was employed to estimate cross-sectional networks for T1 and T2. Network visualization was implemented through the qgraph package (version 1.9.2) (Epskamp et al., 2012), with node-edge diagrams generated using the Fruchterman–Reingold algorithm. To characterize nodal centrality, we computed expected influence (EI) indices for each node, quantifying its overall impact on network connectivity. Given the bipartite structure comprising depressive and problematic smartphone use symptom communities, bridging expected influence (BEI) was additionally calculated to assess cross-community connectivity. Elevated BEI values indicate symptoms that potentially mediate comorbidity development through inter-community activation (Jones et al., 2021).

Network accuracy and stability were systematically evaluated using the bootnet package (version 1.5) (McNally, 2021). First, the accuracy of the edge weights was determined by plotting the 95% confidence intervals for each edge in the given network, with narrower CIs indicating higher precision. Second, case-dropping bootstrap procedures (using 2,000 bootstrap samples) were applied to establish centrality stability (CS) coefficients. Following established benchmarks, CS values >0.25 and >0.50 were interpreted as indicating acceptable and excellent stability, respectively (Epskamp et al., 2018). Finally, bootstrap difference tests were performed to statistically compare edge weights and centrality indices across networks.

#### 2.3.2 Network comparison

The network comparison test (NCT), a permutation-based statistical approach, was implemented to evaluate structural differences between estimated network models (van Borkulo et al., 2022). This method systematically characterizes network variability through two primary dimensions: global strength invariance and network structure invariance. Global strength invariance assesses the difference in the sum of all edge weights between networks, where higher values indicate stronger overall connectivity among symptoms. Network structure invariance examines the distributional differences in edge weights across networks. At the local level, NCT evaluates specific differences through pairwise comparisons of individual edge weights and nodal centrality metrics, including expected influence and bridging expected influence values. We applied the Bonferroni–Holm correction method to maintain strict control over the family-wise errors. All analyses were conducted using the NetworkComparisonTest package (version 2.2.1) (van Borkulo et al., 2015).

#### 2.3.3 Cross-lagged panel network analysis

The CLPN analysis was estimated using the glmnet package through the following analytical procedures. First, least absolute shrinkage and selection operator (LASSO) regularization with 10-fold cross-validation was implemented to optimize the estimation of autoregressive and cross-lagged edges (Wysocki et al., 2022). Autoregressive effects quantified the stability of symptoms from T1 to T2, calculating the node's self-prediction at T2, adjusted for all other T1 nodes. Cross-lagged paths represented directional influences between distinct nodes across timepoints, specifically capturing how a T1 node predicted its connected T2 node while controlling for autoregressive effects and concurrent symptom associations (Wysocki et al., 2022; Ye et al., 2025).

Two complementary centrality metrics were employed to identify core drivers of network dynamics: out-expected influence (Out-EI) and in-expected influence (In-EI) (Hu et al., 2025; Wang et al., 2025). Out-EI characterizes a symptom's capacity to influence other symptoms, whereas In-EI reflects its susceptibility to external influences. Edge-weight confidence intervals (95% CIs) and CS coefficients for both Out-EI and In-EI were derived through bootstrap resampling (using 2,000 bootstrap samples).

## 3 Results

### 3.1 Demographic characteristics

The final sample comprised 584 medical graduate students (Mean age = 30.47 years, SD = 3.57), with a gender distribution of 381 males (65.2%) and 203 females (34.8%). The demographic characteristics show that 348 participants (59.6%) were unmarried, among whom 185 (31.7%) considered themselves to be single. Nearly half of the sample (*n* = 298, 51.0%) were the only child in their families, and the majority of the participants lived in urban areas (69.2%). In terms of health status, 72.4% of the participants perceived themselves to be in good physical condition. Table 1 presents the descriptive statistics for depressive symptoms and PSU scores across both timepoints, along with standardized abbreviations for each symptom item in the networks.

**Table 1:** Item labels and descriptive statistics of depression symptoms and problematic smartphone use at two-time points.

Item	Label	Time point 1		Time point 2	
*N* = 584		*M*	SD	*M*	SD
Anhedonia	PHQ1	0.36	0.61	0.28	0.54
Depressed or sad mood	PHQ2	0.31	0.54	0.23	0.49
Sleep difficulties	PHQ3	0.40	0.66	0.28	0.55
Fatigue	PHQ4	0.54	0.68	0.38	0.61
Appetite changes	PHQ5	0.29	0.56	0.24	0.58
Feeling of worthlessness	PHQ6	0.20	0.48	0.14	0.40
Concentration difficulties	PHQ7	0.23	0.52	0.18	0.47
Psychomotor agitation/retardation	PHQ8	0.09	0.34	0.08	0.32
Thoughts of death	PHQ9	0.04	0.27	0.04	0.23
Missing planned work	PSU1	2.50	1.37	2.32	1.32
Hard concentrating	PSU2	2.28	1.30	2.12	1.26
Feeling pain	PSU3	2.04	1.23	1.97	1.24
Can't stand not having a phone	PSU4	3.05	1.67	2.70	1.64
Impatient without phone	PSU5	2.39	1.38	2.15	1.33
Having phone in mind	PSU6	1.93	1.18	1.82	1.10
Never give up using a phone	PSU7	2.31	1.43	2.09	1.37
Afraid to miss conversations	PSU8	2.57	1.43	2.32	1.39
Longer than intended	PSU9	2.75	1.43	2.49	1.46
Use the phone too much	PSU10	1.77	1.02	1.78	1.07

M, mean; SD, standard deviation.

### 3.2 Contemporaneous networks

The results of node redundancy detection indicate that PSU7 and PSU5 at T1, PHQ5 and PHQ3 at T2, PSU10 and PSU1 may be redundant node pairs, and there is no potential redundancy between the two symptom communities. Given the unique significance of the above items, we still maintain the original number of items (Everaert and Joormann, 2019).

**Figure 1:**
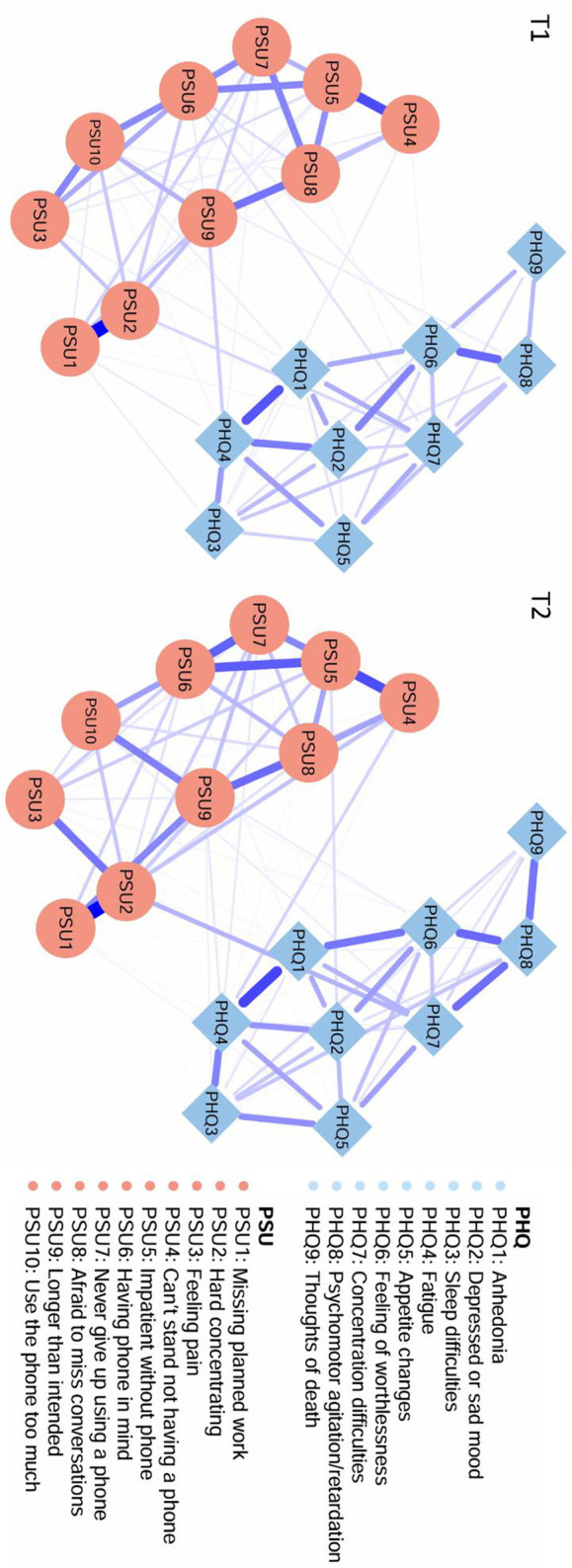
Contemporaneous networks of depressive and PSU symptoms at T1 and T2. The blue edge indicates a positive correlation, and the thickness of the edge reflects the magnitude of the correlation.

**Figure 2:**
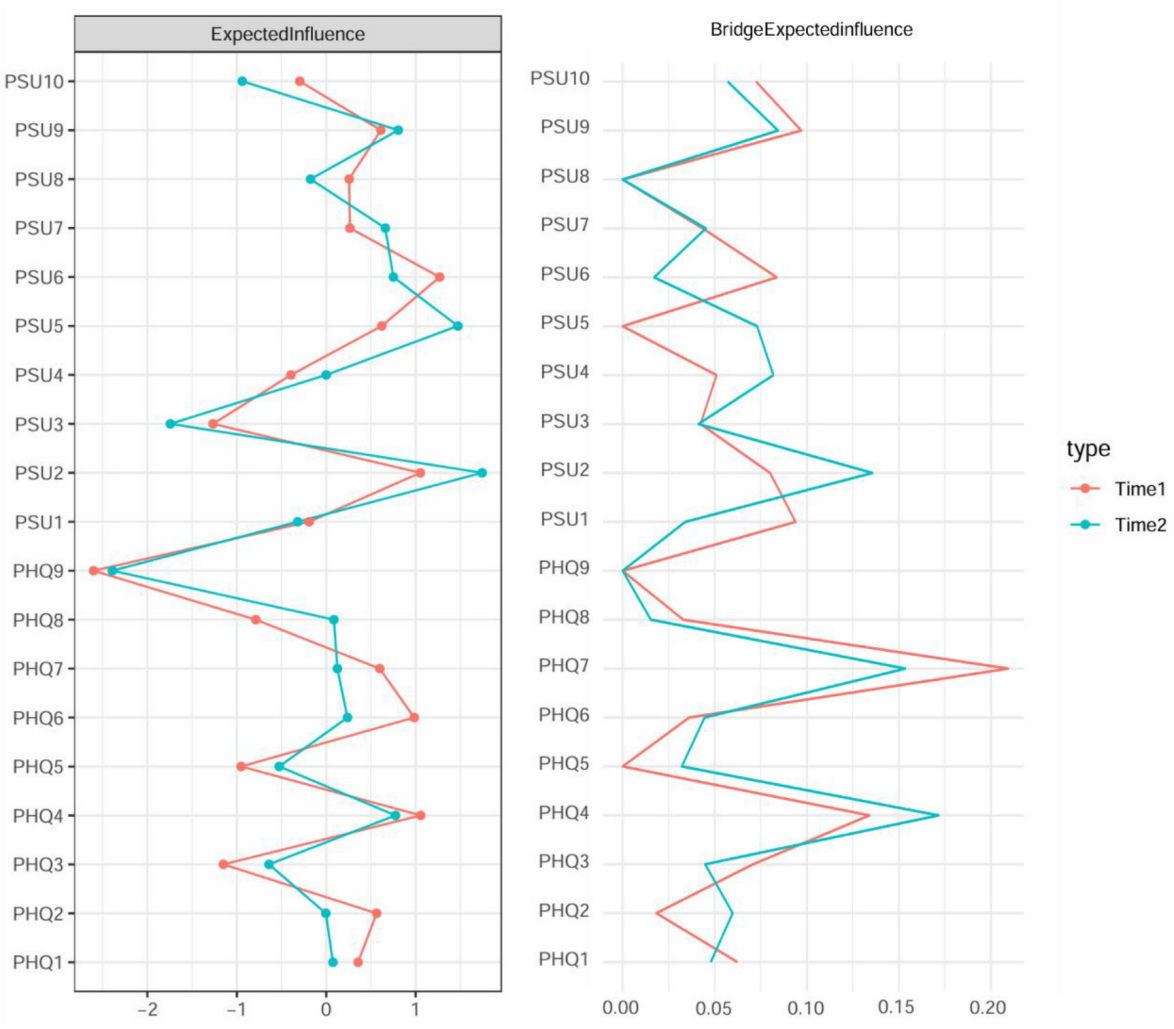
Estimates of expected influence and bridge expected influence (1-step) in the contemporaneous networks at T1 and T2 time points. Standardized *z*-scores are plotted for ease of interpretation. Higher scores represent higher centrality estimates. The text of depression and PSU symptoms can be seen in Table 1.

Figure 1 shows two contemporaneous networks at time points T1 and T2. As shown in Supplementary Tables S1, S2, the strongest edge connecting the PSU and PHQ clusters was between PSU9 (“longer than intended”) and PHQ4 (“fatigue”; weight = 0.09) at T1, and between PSU2 (“hard concentrating”) and PHQ7 (“concentration difficulties”; weight = 0.13) at T2. Centrality analysis based on EI identified key drivers within the network (Figure 2). At T1, PSU6 (“having phone in mind”) exhibited the highest EI, surpassing 55.6% of the remaining nodes. By T2, PSU2 (“hard concentrating”) emerged as the most influential node, exceeding 94.4% of remaining nodes (Supplementary Figure S1). Bridge centrality analysis identified nodes critical for connecting the PSU and PHQ communities. At T1, PHQ7 (“concentration difficulties”) and PHQ4 (“fatigue”) displayed the highest BEI values (0.21 and 0.13, respectively; Figure 2). These values exceeded 94.4% and 22.2% of other nodes. At T2, PHQ4 (“fatigue”) and PHQ7 (“concentration difficulties”) are still identified as bridge symptoms (0.17 and 0.15, respectively; Figure 2), with BEI values exceeding the remaining 83.3% and 72.2% of nodes (Supplementary Figure S2).

The CIs of the edge weights for the networks at the T1 and T2 time points were narrow (Supplementary Figure S3) and significant differences were observed between the strongest and weakest edges (Supplementary Figure S4), indicating that these edges were accurate. Based on the Case-dropping results (Supplementary Figure S5), excellent EI stability (CS = 0.75 for T1 and T2) and good BEI stability (CS = 0.44 for T1 and T2) were shown.

### 3.3 Network comparison

To facilitate a visual comparison of the network structures, the averaged layout was used for both networks. The T1 network had 89 non-zero edges (52.0% of 171 edges) and the T2 network had 93 non-zero edges (54.4% of 171 possible edges). In the comparison test of the two networks, no significant differences were found in terms of network structure (*p* = 0.37), indicating that no edge weights differed significantly across the two samples. The T1 network with a global strength of 8.45 is not significantly different from the T2 network with a global strength of 8.49 (*p* = 0.76). Besides, there were no significant differences concerning nodes' expected influence and bridge expected influence between the networks, the above results indicated that this network exhibited consistency across different time points.

### 3.4 Cross-lagged panel networks

**Figure 3:**
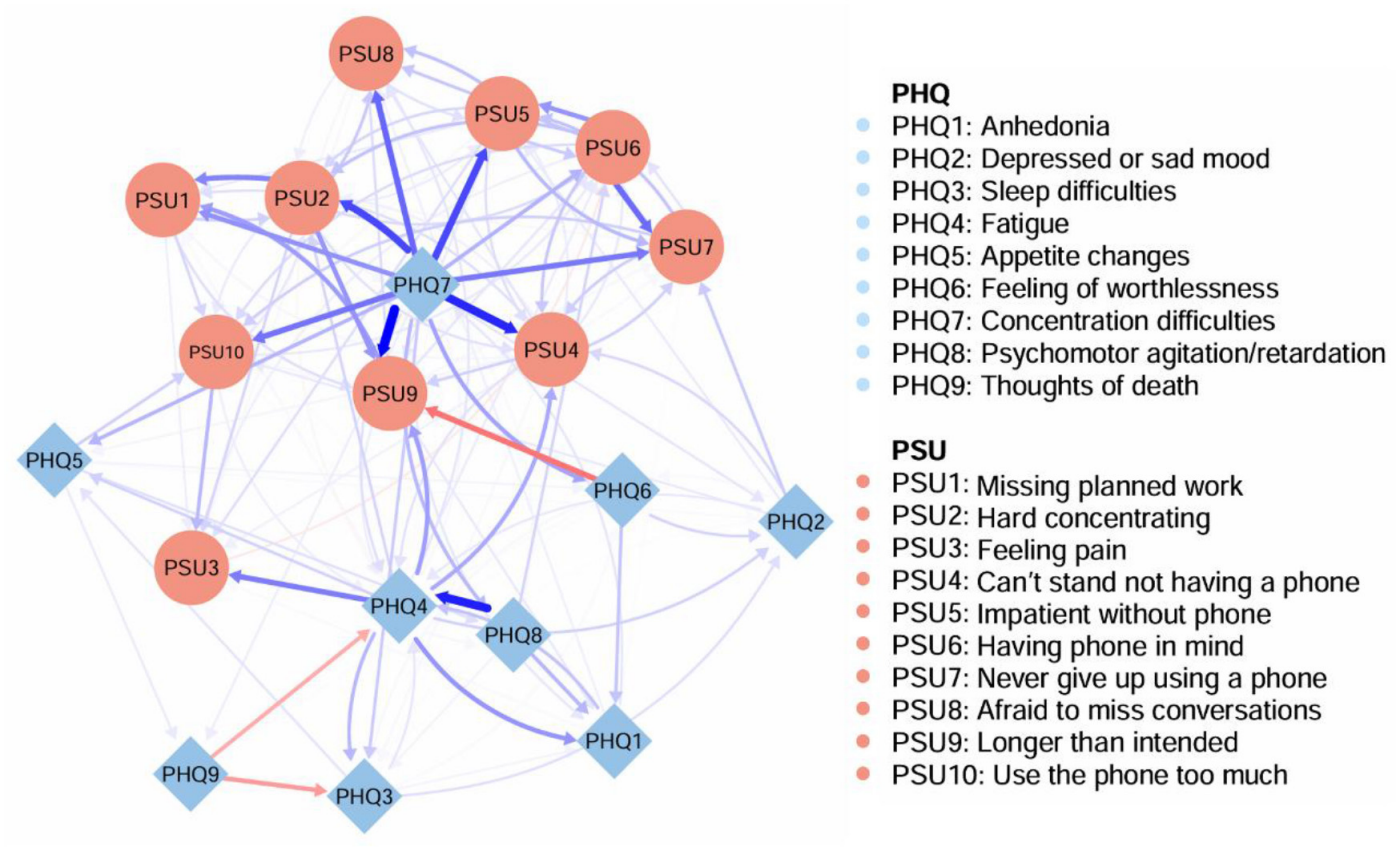
The cross-lagged panel network of depressive and PSU symptoms from T1 to T2. Arrows represent unique longitudinal predictive relationships. Blue edges indicate positive relationships, and red edges indicate negative relationships.

Figure 3 shows the CLPN from T1 to T2. In this network 135 directed cross-lagged edges and 17 autoregressive edges were shown. Supplementary Table S3 shows the full adjacency matrix with edge weights represented by partial directed correlations in the CLPN network. In the CLPN we constructed, most edges were positive, yet a small number of negative predictive edges were still present. For instance, “suicidal ideation” among depressive symptoms negatively predicted “sleep difficulties” and “fatigue” symptoms. For within-disorder edges, the strongest three were: PSU6 (“having phone in mind”) → PSU7 (“never give up using a phone”; weight = 0.21), PSU2 (“hard concentrating”) → PSU1 (“missing planned work”; weight = 0.18) and PHQ8 (“psychomotor agitation/retardation”) → PHQ4 (“fatigue”; weight = 0.33). The strongest edges connecting depressive and PSU symptoms all directed from PHQ7 (“concentration difficulties”) to PSU symptoms: PHQ7 (“concentration difficulties”) → PSU9 (“longer than intended”; weight = 0.37), PHQ7 (“concentration difficulties”) → PSU4 (“can't stand not having a phone”; weight = 0.32), PHQ7 (“Concentration difficulties”) → PSU5 (“Impatient without phone”; weight = 0.27) and PHQ7 (“concentration difficulties”) → PSU2 (“hard concentrating”; weight = 0.27). Further analysis of the results revealed that almost all edges connecting depression and PSU symptoms directed from depressive symptoms to PSU symptoms, and that PHQ7 was associated with a variety of subsequent PSU symptoms, suggesting that it was the strongest predictor of PSU symptoms.

**Figure 4:**
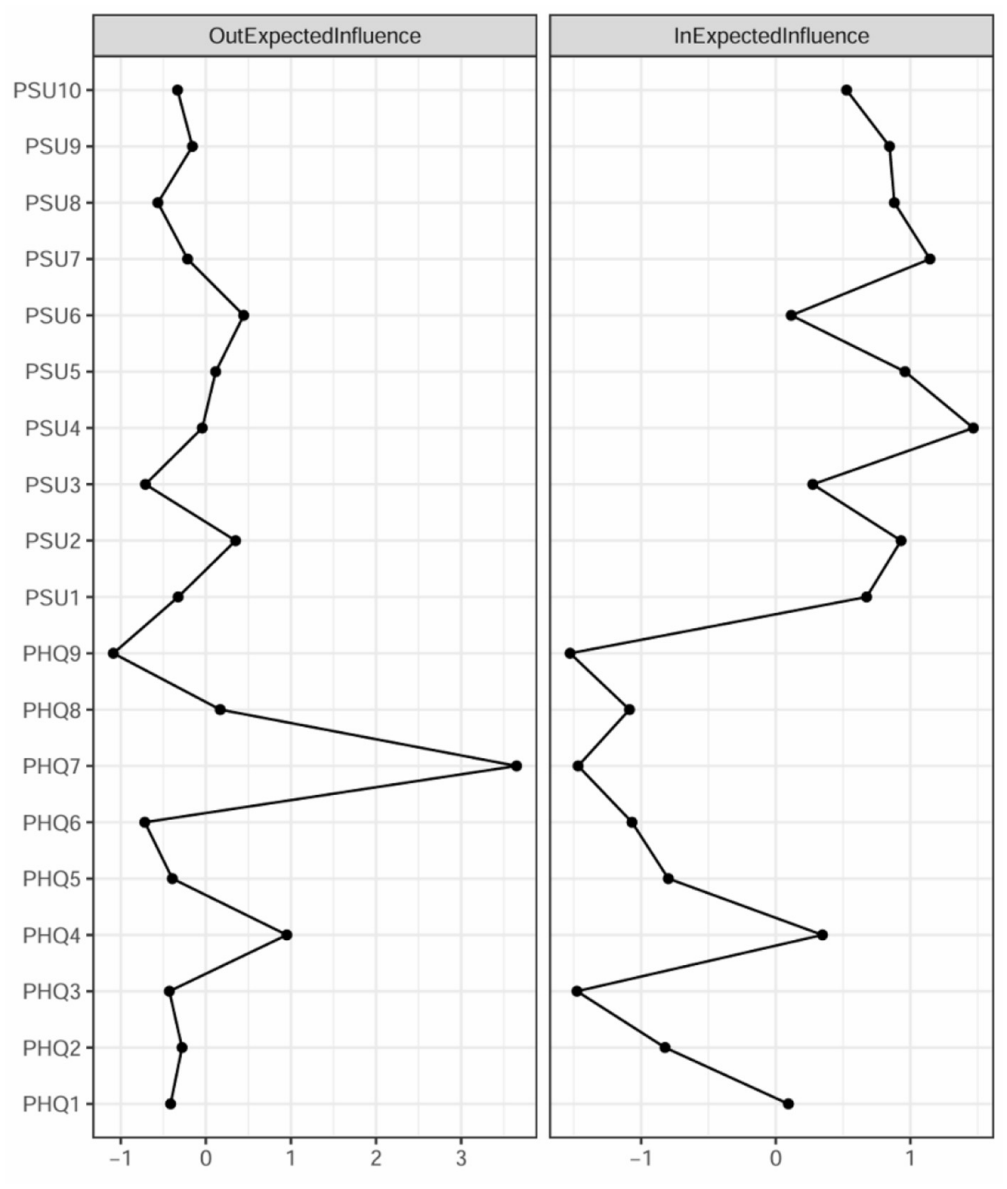
Out-expected influence and in-expected influence values in the CLPN. Standardized *z*-scores are plotted for ease of interpretation. Higher scores represent higher centrality estimates. The text of depression and PSU symptoms can be seen in Table 1.

**Figure 5:**
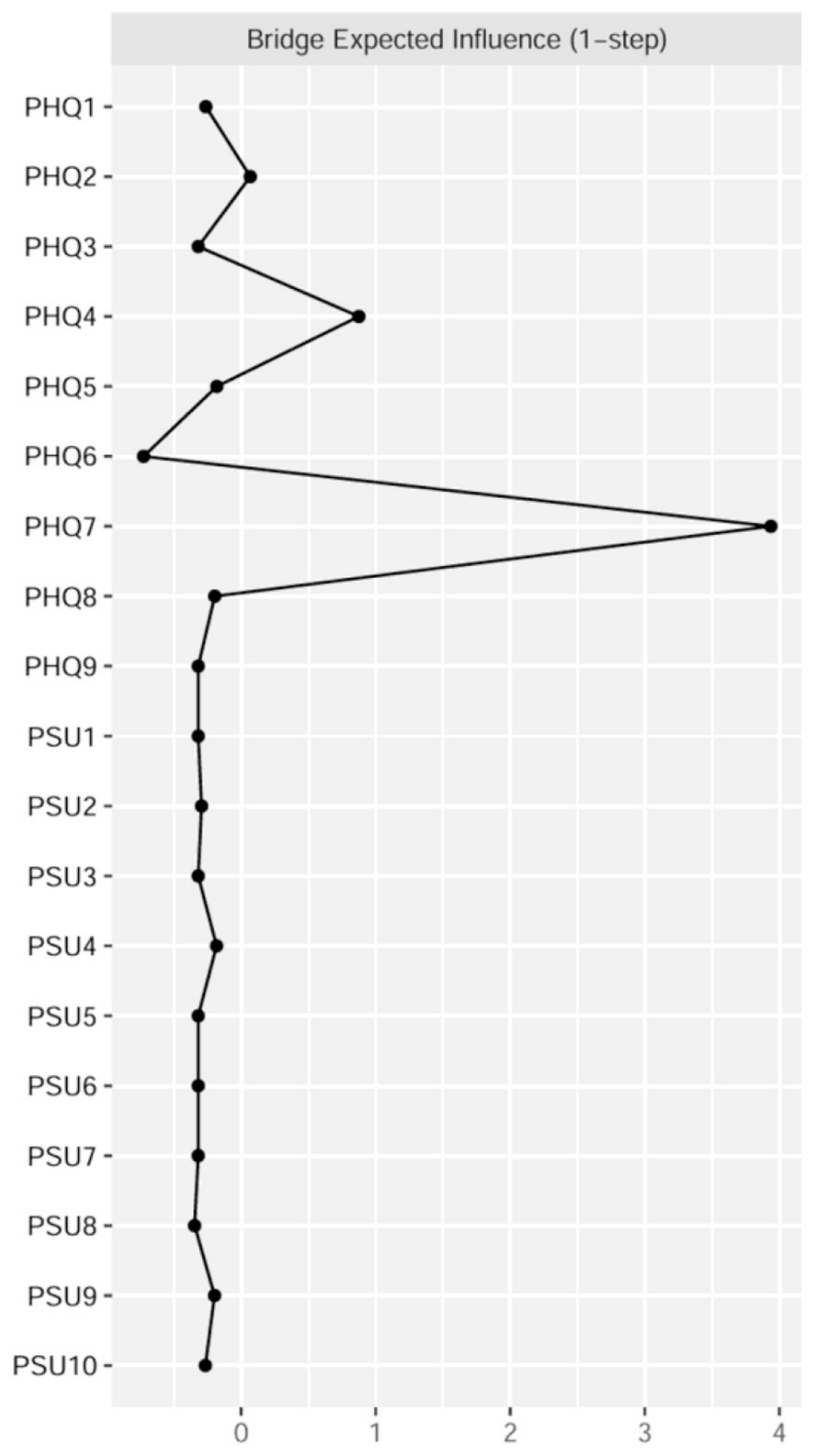
Bridge expected influence values in the CLPN. Larger values reflect greater centrality. The text of depression and PSU symptoms can be seen in Table 1.

Figure 4 shows the out-EI and in-EI estimates of CLPN network. PHQ7 (“concentration difficulties”) and PHQ4 (“fatigue”) had the highest out-EI (3.65, 0.95; significantly surpassing than most of the residual nodes in the network; see Supplementary Figure S6) and low in-EI, suggesting they were the strongest predictors for other depressive symptoms and PSU symptoms. PSU4 (“can't stand not having a phone”) and PSU7 (“never give up using a phone”) had the highest in-EI (1.47; 1.14) and low out-EI, indicating they were more likely to be triggered by other symptoms rather than activating the CLPN network. Based on the results of Bridge expected influence (Figure 5), PHQ7 (“concentration difficulties”) and PHQ4 (“fatigue”) exhibited the highest BEI values (2.11; 0.59), indicating they exerted the strongest influence on the symptoms within the PSU community.

The CIs around the edge weights of the network were relatively narrow (Supplementary Figure S7) and the strongest edges were significantly different from the weakest edges (Supplementary Figure S8), indicating that these edges were accurate. According to the case-dropping results (Supplementary Figure S9), the stability was acceptable for the bridge-EI (CS coefficient = 0.28), in-EI (CS coefficient = 0.75), and out-EI (CS coefficient = 0.28).

## 4 Discussion

This study employed CLPN model to investigate the longitudinal relationships between PSU and depressive symptoms among Chinese medical graduate students. By mapping contemporaneous and temporal symptom-level interactions, we identified dynamic pathways and central mechanisms underlying their comorbidity. As the first longitudinal network analysis focusing on this high-stress population, our findings advance understanding of how specific depressive symptoms drive PSU over time, offering critical insights for targeted interventions. The results highlight the importance of addressing core depressive symptoms to disrupt the PSU-depression cycle, which is particularly relevant for medical graduate students facing unique academic and professional pressures.

Important bridging connections in the co-morbidity network at different time points reflect mechanistic pathways that promote the development of depressive symptoms and PSU. Temporal changes in the bridging edge between the PSU and the depression network reflect the evolution of co-morbid mechanisms (Jia et al., 2024). At T1, the strongest cross-community edge existed between PSU9 (“longer than intended”) and PHQ4 (“fatigue”), consistent with compensatory internet use theory (Kardefelt-Winther, 2014). As this association is cross-sectional within a single time point, it does not permit directional inference. Nevertheless, fatigue is recognized as a sign of chronic academic stress, and medical students experiencing fatigue may turn to prolonged smartphone use as a maladaptive coping mechanism to escape cognitive overload (Wang et al., 2023). However, this type of regulation requires substantial compensation to alleviate negative emotions, and such a habit may lead to negative consequences and addiction symptoms in the long term, resulting in more uncontrollable mobile phone use and increased psychological fatigue due to depletion of cognitive resources (Kardefelt-Winther, 2014). In addition, it has been found that sleep disorders in medical students mediate the relationship between PSU and daytime fatigue (Zhang et al., 2021), suggesting that PSU behaviors may increase fatigue by affecting sleep quality. By T2, the strongest bridging edges shifted to PSU2 (“hard concentrating”) and PHQ7 (“concentration difficulties”), indicating a transition from physical fatigue to cognitive impairment as the dominant pathway. The temporal shift suggests that early intervention targeting fatigue, for example by limiting mobile phone use, can prevent downstream cognitive dysfunction and PSU escalation.

PHQ7 (“concentration difficulties”) and PHQ4 (“fatigue”) were identified as bridging symptoms in both contemporaneous networks, suggesting that they may play an important role in contributing to the development of symptoms and co-morbidity formation across different points (Zhao et al., 2023). Although both PHQ7 (“concentration difficulties”) and PHQ4 (“fatigue”) were the primary bridging symptoms, their effects varied dynamically across time points. At T1, PHQ7 showed the highest degree of bridging centrality, and concentration difficulties may prompt vicarious use of a smartphone for temporary distraction, thus unintentionally reinforcing dependence (Tao et al., 2024). Such vicarious behavior, in turn, directly interferes with academic progress and clinical work, thereby increasing core stressors for medical students (Sohn et al., 2019). This result was consistent with previous studies that identified attention deficit as a key bridging node between PSU and depressive symptoms (Guo et al., 2023; Wei et al., 2023). However, the previous studies were cross-sectional and could not provide directional relationships between different communities. At T2, PHQ-4 (“fatigue”) exhibited a higher BEI value than PHQ-7 (“concentration difficulties”). This finding aligns with a recent temporal network study identifying fatigue as a key bridge symptom linking PSU and depression (Hu et al., 2025). Given the combined demands of research and clinical rotations—factors that may contribute to elevated fatigue—this pattern may hold particular relevance for medical graduate students. The shift in bridge centrality underscores the need for stage-specific interventions, and early intervention in cognitive resilience training for attentional control may be more effective from a bridge centrality perspective.

The CLPN network revealed that nearly all bridging pathways flowed from depressive symptoms to PSU symptoms, which is consistent with the previously proposed I-PACE model and the theory of compensatory internet use (Brand et al., 2019; Kardefelt-Winther, 2014). This finding is consistent with previous research, which identified reciprocal associations between anxiety symptoms and problematic smartphone use, with symptoms forming a vicious cycle over time in which anxiety symptoms played a more prominent role (Wang et al., 2025). Together, these findings support the emotion regulation theory, which posits that individuals use smartphones to alleviate negative emotions and reduce cognitive fatigue (Thomée et al., 2011). Smartphones, as the most commonly used portable internet device, will undoubtedly become the preferred choice of students for relaxation and entertainment, as it may increase the level of dependence on them and have an adverse effect on mental health and academic performance (Amin et al., 2024; Grant et al., 2019). Notably, a previous study examining the bidirectional relationship between problematic internet use and depression among Chinese college students suggested that comorbidity mechanisms and core symptoms may change over time (Jia et al., 2024). Building on this finding, future research could incorporate data from different academic years to more precisely delineate the impact of academic stress on comorbidity mechanisms across stages. Overall, our findings suggest that depressive symptoms may play a significant activating role in the development of problematic smartphone use, highlighting the importance of investigating comorbidity mechanisms within specific populations.

PHQ7 (“concentration difficulties”) showed the highest out-EI in the CLPN network and robustly predicted multiple PSU symptoms, covering a variety of symptoms. This aligns with the I-PACE model, where impaired executive control (e.g., attention deficits) heightens susceptibility to impulsive smartphone use (Brand et al., 2016). Medical students struggling to focus during intensive study may develop habitual phone-checking as a cognitive reset mechanism, inadvertently reinforcing dependency. The results of the present study validate and extend the cross-sectional findings that concentration difficulties serve the most important role in the development and activation of PSU (Guo et al., 2023; Wei et al., 2023). Studies have confirmed that college students' self-perceived attention deficit and hyperactivity disorder significantly affect the level of smartphone addiction (Zeyrek et al., 2024). The portability of smartphones and the instant response and rewards they can provide make them more attractive to groups that have difficulty concentrating. Deficits in self-control and inhibition can increase difficulties with excessive smartphone use and exacerbate ADHD symptoms (Serra et al., 2021). In addition, recent research has also shown that people with difficulties in attentional control are more likely to develop mental health problems when using social media, which also supports our findings (Mahalingham et al., 2022).

The directed connectivity of PHQ7 to multiple depressive symptoms and its highest out-EI values also provide more reliable evidence for the results of the concurrent network, supporting that PHQ7 (“concentration difficulties”) has the strongest activating effect on subsequent depressive symptoms. Furthermore, by integrating different centrality indicators, we found that PHQ4 (“fatigue”) exerted a profound impact on PSU symptoms, which is consistent with a recent study on Chinese adolescents (Hu et al., 2025). This directional interpretation is supported by our longitudinal CLPN results: PHQ4 at T1 positively predicted PSU9 at T2 (partial directed correlation = 0.14), whereas the reverse path (PSU9 at T1 to PHQ4 at T2) was considerably weaker (0.03). These temporal findings suggest that fatigue may drive subsequent increases in uncontrolled smartphone use duration. One plausible explanation is that smartphones are often used as an important means of relaxation, with individuals seeking pleasure and relief from fatigue through various social media platforms on their phones (Abuhamdah and Naser, 2023). Targeted interventions addressing these specific symptomatic features can not only effectively and precisely alleviate problematic smartphone use behaviors and depressive symptoms among medical graduate students but also potentially prevent the emergence and development of comorbid psychiatric disorders. Interventions focusing on attentional control, such as mindfulness-based cognitive therapy (MBCT), can block this pathway by enhancing metacognitive awareness of problematic smartphone use patterns (Kim et al., 2024).

### 4.1 Limitations and future directions

Although this study advances the understanding of the co-morbidity dynamics of PSU and depression in medical graduate students, there are some limitations worth noting. First, this study included a non-clinical sample population, and future studies should extend to clinical samples to further validate the applicability of our conclusions. Second, the two-wave design of this study limits the ability to examine long-term trajectories of symptom evolution. Given the extended duration of medical education, this issue warrants further investigation in future research. Subsequent studies may consider collecting data across multiple academic years to more comprehensively clarify the influence of academic stress on comorbidity mechanisms and core symptoms at different stages (Zhang et al., 2020). Third, self-reported data may introduce recall bias, and future research should incorporate objective metrics of smartphone use (e.g., screen time tracking) and incorporate physiological markers to further elucidate biopsychological mechanisms. Fourth, we recruited the sample from two medical universities through purposeful recruitment, which limits generalizability to populations with different academic pressures. Finally, the present findings supported that specific depressive symptoms may contribute to subsequent problematic smartphone use, which is closely related to the study's temporal parameters and individuals' motivational patterns of smartphone engagement. Future research should focus on the specific motivations and patterns of mobile phone use (Liu et al., 2019), to further elucidate the complex dynamics between PSU and depression symptoms.

## 5 Conclusion

This longitudinal network analysis study delineated the complex dynamic associations between PSU and depression among Chinese medical postgraduates at the symptom level. Network comparisons revealed that the PSU-depression network showed overall consistency across time points, and that PHQ7 (“concentration difficulties”) and PHQ4 (“fatigue”) were identified as key bridging symptoms contributing to the development of co-morbidity at two time points. The CLPN results revealed that comorbidities predominantly develop from depression to PSU, with PHQ7 (“concentration difficulties”) having the strongest activating effect on subsequent PSU symptoms. PSU4 (“can't stand not having a phone”) and PSU7 (“never give up using a phone”) presented the highest in-EI, indicating they were most susceptible to other nodes. Identifying these specific symptoms can guide precise interventions at the symptom level, advocate for early interventions targeting attentional control and fatigue management, and enhance mental health and professional competence of future healthcare providers.

## Data Availability

The raw data supporting the conclusions of this article will be made available by the authors, without undue reservation.
