# Supplementary material for: Depressive symptoms precede and drive problematic smartphone use in Chinese medical students: a longitudinal network analysis
Source: Front Psychol. 2026 May 8;17:1808905. doi: 10.3389/fpsyg.2026.1808905 (PMC13194431; doi:10.3389/fpsyg.2026.1808905)
Supplement: Supplementary file 1 [file Table_1.docx]

**Supplementary materials**

**The longitudinal relationships between problematic smartphone use and depression symptoms among Chinese medical graduate students: A cross-lagged panel network analysis**

Table S1. Partial correlation coefficients between undirected edges at T1 network

Table S2. Partial correlation coefficients between undirected edges at T2 network

Table S3. Adjacency matrix of the T1 → T2 cross-lagged panel network

Figure S1. Bootstrapped difference tests for the node’s expected influence of the contemporaneous networks across Time 1 to Time 2

Figure S2. Bootstrapped difference tests for the node’s bridge expected influence of the contemporaneous networks across Time 1 to Time 2

Figure S3*.* Accuracy of the edge estimates of the contemporaneous networks across Time 1 to Time 2

Figure S4. Bootstrapped difference tests (α = 0.05) between edges of the contemporaneous networks across Time 1 to Time 2

Figure S5. Stability of central indices of the contemporaneous networks across Time 1 to Time 2

Figure S6. Bootstrapped difference tests for the node’s out-expected influence, in-expected influence and bridge expected influence of the cross-lagged panel network

Figure S7. Accuracy of the edge estimates of the cross-lagged panel networks

Figure S8. Bootstrapped difference tests (α = 0.05) between edges of the cross-lagged panel network

Figure S9. Stability of central indices of the cross-lagged panel network

Table S1. Partial correlation coefficients between undirected edges at T1 network

| Time 1 | PHQ1 | PHQ2 | PHQ3 | PHQ4 | PHQ5 | PHQ6 | PHQ7 | PHQ8 | PHQ9 | PSU1 | PSU2 | PSU3 | PSU4 | PSU5 | PSU6 | PSU7 | PSU8 | PSU9 | PSU10 |
| --- | --- | --- | --- | --- | --- | --- | --- | --- | --- | --- | --- | --- | --- | --- | --- | --- | --- | --- | --- |
| PHQ1 | — |  |  |  |  |  |  |  |  |  |  |  |  |  |  |  |  |  |  |
| PHQ2 | 0.17 | — |  |  |  |  |  |  |  |  |  |  |  |  |  |  |  |  |  |
| PHQ3 | 0.03 | 0.13 | — |  |  |  |  |  |  |  |  |  |  |  |  |  |  |  |  |
| PHQ4 | 0.32 | 0.25 | 0.19 | — |  |  |  |  |  |  |  |  |  |  |  |  |  |  |  |
| PHQ5 | 0.05 | 0.03 | 0.09 | 0.19 | — |  |  |  |  |  |  |  |  |  |  |  |  |  |  |
| PHQ6 | 0.17 | 0.23 | 0.01 | 0.00 | 0.07 | — |  |  |  |  |  |  |  |  |  |  |  |  |  |
| PHQ7 | 0.15 | 0.08 | 0.10 | 0.00 | 0.16 | 0.11 | — |  |  |  |  |  |  |  |  |  |  |  |  |
| PHQ8 | 0.00 | 0.06 | 0.00 | 0.00 | 0.12 | 0.29 | 0.11 | — |  |  |  |  |  |  |  |  |  |  |  |
| PHQ9 | 0.00 | 0.02 | 0.06 | 0.00 | 0.00 | 0.14 | 0.06 | 0.13 | — |  |  |  |  |  |  |  |  |  |  |
| PSU1 | 0.01 | 0.00 | 0.03 | 0.04 | 0.00 | 0.00 | 0.02 | 0.00 | 0.00 | — |  |  |  |  |  |  |  |  |  |
| PSU2 | 0.00 | 0.00 | 0.00 | 0.00 | 0.00 | 0.00 | 0.08 | 0.00 | 0.00 | 0.49 | — |  |  |  |  |  |  |  |  |
| PSU3 | 0.02 | 0.00 | 0.00 | 0.00 | 0.00 | 0.00 | 0.01 | 0.00 | 0.00 | 0.00 | 0.11 | — |  |  |  |  |  |  |  |
| PSU4 | 0.00 | 0.00 | 0.03 | 0.00 | 0.00 | 0.00 | 0.02 | 0.00 | 0.00 | 0.00 | 0.01 | 0.03 | — |  |  |  |  |  |  |
| PSU5 | 0.00 | 0.00 | 0.00 | 0.00 | 0.00 | 0.00 | 0.00 | 0.00 | 0.00 | 0.04 | 0.03 | 0.07 | 0.34 | — |  |  |  |  |  |
| PSU6 | 0.00 | 0.00 | 0.00 | 0.00 | 0.00 | 0.03 | 0.05 | 0.00 | 0.00 | 0.00 | 0.10 | 0.16 | 0.00 | 0.23 | — |  |  |  |  |
| PSU7 | 0.00 | 0.00 | 0.00 | 0.02 | 0.00 | 0.00 | 0.02 | 0.00 | 0.00 | 0.09 | 0.03 | 0.01 | 0.15 | 0.08 | 0.22 | — |  |  |  |
| PSU8 | 0.00 | 0.00 | 0.00 | 0.00 | 0.00 | 0.00 | 0.00 | 0.00 | 0.00 | 0.00 | 0.02 | 0.00 | 0.12 | 0.21 | 0.07 | 0.23 | — |  |  |
| PSU9 | 0.00 | 0.00 | 0.00 | 0.09 | 0.00 | 0.00 | 0.00 | 0.00 | 0.00 | 0.11 | 0.12 | 0.01 | 0.12 | 0.00 | 0.03 | 0.09 | 0.27 | — |  |
| PSU10 | 0.03 | 0.01 | 0.00 | -0.02 | 0.00 | 0.01 | 0.01 | 0.03 | 0.00 | 0.04 | 0.10 | 0.23 | 0.00 | 0.00 | 0.22 | 0.00 | 0.02 | 0.15 | — |

*Note.* PHQ1 = Anhedonia, PHQ2 = Depressed or sad mood, PHQ3 = Sleep difficulties, PHQ4 = Fatigue, PHQ5 = Appetite changes, PHQ6 = Feeling of worthlessness, PHQ7 = Concentration difficulties, PHQ8 = Psychomotor agitation/retardation, PHQ9 = Thoughts of death, PSU1 = Missing planned work, PSU2 = Hard concentrating, PSU3 = Feeling pain, PSU4 = Can’t stand not having a phone, PSU5 = Impatient without phone, PSU6 = Having phone in mind, PSU7 = Never give up using a phone, PSU8 = Afraid to miss conversations, PSU9 = Longer than intended, PSU10 = Use the phone too much.

Table S2. Partial correlation coefficients between undirected edges at T2 network

| Time 2 | PHQ1 | PHQ2 | PHQ3 | PHQ4 | PHQ5 | PHQ6 | PHQ7 | PHQ8 | PHQ9 | PSU1 | PSU2 | PSU3 | PSU4 | PSU5 | PSU6 | PSU7 | PSU8 | PSU9 | PSU10 |
| --- | --- | --- | --- | --- | --- | --- | --- | --- | --- | --- | --- | --- | --- | --- | --- | --- | --- | --- | --- |
| PHQ1 | — |  |  |  |  |  |  |  |  |  |  |  |  |  |  |  |  |  |  |
| PHQ2 | 0.14 | — |  |  |  |  |  |  |  |  |  |  |  |  |  |  |  |  |  |
| PHQ3 | 0.04 | 0.10 | — |  |  |  |  |  |  |  |  |  |  |  |  |  |  |  |  |
| PHQ4 | 0.31 | 0.17 | 0.21 | — |  |  |  |  |  |  |  |  |  |  |  |  |  |  |  |
| PHQ5 | 0.00 | 0.11 | 0.18 | 0.15 | — |  |  |  |  |  |  |  |  |  |  |  |  |  |  |
| PHQ6 | 0.23 | 0.16 | 0.03 | 0.01 | 0.10 | — |  |  |  |  |  |  |  |  |  |  |  |  |  |
| PHQ7 | 0.12 | 0.06 | 0.01 | 0.02 | 0.16 | 0.10 | — |  |  |  |  |  |  |  |  |  |  |  |  |
| PHQ8 | 0.02 | 0.05 | 0.10 | 0.00 | 0.01 | 0.25 | 0.24 | — |  |  |  |  |  |  |  |  |  |  |  |
| PHQ9 | 0.00 | 0.04 | 0.06 | 0.00 | 0.04 | 0.01 | 0.06 | 0.23 | — |  |  |  |  |  |  |  |  |  |  |
| PSU1 | 0.01 | 0.00 | 0.00 | 0.01 | 0.02 | 0.00 | 0.00 | 0.00 | 0.00 | — |  |  |  |  |  |  |  |  |  |
| PSU2 | 0.01 | 0.00 | 0.00 | 0.00 | 0.00 | 0.00 | 0.13 | 0.00 | 0.00 | 0.42 | — |  |  |  |  |  |  |  |  |
| PSU3 | 0.01 | 0.00 | 0.01 | 0.00 | 0.00 | 0.00 | 0.00 | 0.02 | 0.00 | 0.00 | 0.23 | — |  |  |  |  |  |  |  |
| PSU4 | 0.00 | 0.00 | 0.01 | 0.07 | 0.00 | 0.00 | 0.00 | 0.00 | 0.00 | 0.10 | 0.02 | 0.01 | — |  |  |  |  |  |  |
| PSU5 | 0.00 | 0.06 | 0.01 | 0.01 | 0.00 | 0.00 | 0.00 | 0.00 | 0.00 | 0.00 | 0.08 | 0.08 | 0.29 | — |  |  |  |  |  |
| PSU6 | 0.02 | 0.00 | 0.00 | 0.00 | 0.00 | 0.00 | 0.00 | 0.00 | 0.00 | 0.08 | 0.05 | 0.08 | 0.00 | 0.27 | — |  |  |  |  |
| PSU7 | 0.00 | 0.00 | 0.00 | 0.04 | 0.01 | 0.00 | 0.00 | 0.00 | 0.00 | 0.00 | 0.08 | 0.04 | 0.12 | 0.20 | 0.26 | — |  |  |  |
| PSU8 | 0.00 | 0.00 | 0.00 | 0.00 | 0.00 | 0.00 | 0.00 | 0.00 | 0.00 | 0.00 | 0.05 | 0.00 | 0.15 | 0.15 | 0.12 | 0.09 | — |  |  |
| PSU9 | 0.00 | 0.00 | 0.02 | 0.04 | 0.01 | 0.02 | 0.00 | 0.00 | 0.00 | 0.20 | 0.05 | 0.03 | 0.12 | 0.00 | 0.00 | 0.10 | 0.24 | — |  |
| PSU10 | 0.00 | 0.00 | 0.00 | 0.00 | 0.00 | 0.03 | 0.03 | 0.00 | 0.00 | 0.00 | 0.09 | 0.05 | 0.00 | 0.01 | 0.16 | 0.08 | 0.06 | 0.21 | — |

*Note.* PHQ1 = Anhedonia, PHQ2 = Depressed or sad mood, PHQ3 = Sleep difficulties, PHQ4 = Fatigue, PHQ5 = Appetite changes, PHQ6 = Feeling of worthlessness, PHQ7 = Concentration difficulties, PHQ8 = Psychomotor agitation/retardation, PHQ9 = Thoughts of death, PSU1 = Missing planned work, PSU2 = Hard concentrating, PSU3 = Feeling pain, PSU4 = Can’t stand not having a phone, PSU5 = Impatient without phone, PSU6 = Having phone in mind, PSU7 = Never give up using a phone, PSU8 = Afraid to miss conversations, PSU9 = Longer than intended, PSU10 = Use the phone too much.

Table S3. Partial directed correlations of T1→T2 cross-lagged panel network.

|  | PHQ1 | PHQ2 | PHQ3 | PHQ4 | PHQ5 | PHQ6 | PHQ7 | PHQ8 | PHQ9 | PSU1 | PSU2 | PSU3 | PSU4 | PSU5 | PSU6 | PSU7 | PSU8 | PSU9 | PSU10 |
| --- | --- | --- | --- | --- | --- | --- | --- | --- | --- | --- | --- | --- | --- | --- | --- | --- | --- | --- | --- |
| PHQ1 | 0.07 | 0 | 0 | 0.09 | 0 | 0 | 0.04 | 0 | 0 | 0 | 0 | 0.01 | 0 | 0 | 0 | 0 | 0.02 | 0 | 0 |
| PHQ2 | 0 | 0.18 | 0 | 0.03 | 0 | 0.01 | 0 | 0 | 0 | 0 | 0 | 0 | 0.06 | 0.03 | 0 | 0.08 | 0 | 0 | 0.03 |
| PHQ3 | 0.02 | 0.05 | 0.24 | 0.04 | 0.04 | 0.01 | 0 | 0 | 0 | 0 | 0 | 0 | 0 | 0 | 0 | 0 | 0 | 0 | 0 |
| PHQ4 | 0.15 | 0.07 | 0.1 | 0.22 | 0.06 | 0 | 0.01 | 0.01 | 0 | 0.01 | 0.06 | 0.19 | 0.12 | 0 | 0.03 | 0 | 0.04 | 0.14 | 0 |
| PHQ5 | 0 | 0.01 | 0 | 0 | 0.23 | 0.02 | 0 | 0.04 | 0.03 | 0 | 0 | 0 | 0 | 0 | 0 | 0 | 0 | 0 | 0.07 |
| PHQ6 | 0.09 | 0.06 | 0.01 | 0 | 0 | 0.22 | 0 | 0 | 0 | 0 | 0 | 0 | 0 | 0 | 0 | 0 | 0 | -0.2 | 0 |
| PHQ7 | 0.04 | 0.04 | 0.07 | 0.05 | 0.11 | 0.13 | 0.32 | 0.1 | 0.02 | 0.16 | 0.27 | 0 | 0.32 | 0.27 | 0.11 | 0.19 | 0.22 | 0.37 | 0.2 |
| PHQ8 | 0.11 | 0 | 0.02 | 0.33 | 0 | 0 | 0.01 | 0.04 | 0 | 0 | 0 | 0 | 0 | 0 | 0.06 | 0 | 0 | 0 | 0 |
| PHQ9 | 0 | 0 | -0.15 | -0.12 | 0 | 0 | 0 | 0 | 0 | 0 | 0 | 0 | 0 | 0 | 0 | 0 | 0 | 0 | 0 |
| PSU1 | 0 | 0 | 0 | 0 | 0 | 0 | 0 | 0 | 0 | 0.2 | 0.07 | 0.02 | 0.01 | 0 | 0 | 0 | 0 | 0.05 | 0.06 |
| PSU2 | 0 | 0 | 0 | 0 | 0 | 0 | 0.01 | 0 | 0 | 0.18 | 0.27 | 0.06 | 0.04 | 0.03 | 0.05 | 0 | 0.08 | 0.15 | 0.03 |
| PSU3 | 0 | 0 | 0 | 0 | 0 | 0 | 0 | 0 | 0 | 0 | 0 | 0.12 | 0 | 0 | -0.03 | 0 | 0 | 0 | 0 |
| PSU4 | 0 | 0 | 0.01 | 0.04 | 0.01 | 0 | 0 | 0 | 0 | 0.05 | 0.02 | 0.02 | 0.35 | 0.03 | 0.03 | 0.07 | 0.04 | 0.07 | 0 |
| PSU5 | 0 | 0 | 0 | 0 | 0 | 0 | 0 | 0 | 0 | 0.03 | 0.06 | 0.05 | 0.05 | 0.22 | 0.07 | 0.1 | 0.09 | 0.03 | 0.01 |
| PSU6 | 0 | 0 | 0 | 0 | 0 | 0 | 0 | 0 | 0 | 0 | 0.09 | 0.02 | 0.08 | 0.15 | 0.29 | 0.21 | 0.08 | 0 | 0.05 |
| PSU7 | 0 | 0 | 0 | 0 | 0 | 0 | 0 | 0 | 0 | 0 | 0.01 | 0 | 0.07 | 0.09 | 0.04 | 0.12 | 0 | 0 | 0.07 |
| PSU8 | 0 | 0 | 0 | -0.01 | 0 | 0 | 0 | 0 | 0 | 0 | 0.02 | 0.01 | 0 | 0.04 | 0 | 0 | 0.25 | 0 | 0 |
| PSU9 | 0.03 | 0 | 0 | 0.03 | 0 | 0 | 0 | 0 | 0 | 0.14 | 0 | 0 | 0 | 0 | 0.04 | 0.01 | 0.05 | 0.3 | 0.01 |
| PSU10 | 0 | 0 | 0 | 0.02 | 0 | 0 | 0 | 0 | 0 | 0 | 0.03 | 0.1 | 0 | 0 | 0.03 | 0.01 | 0 | 0 | 0.22 |

*Note.* PHQ1 = Anhedonia, PHQ2 = Depressed or sad mood, PHQ3 = Sleep difficulties, PHQ4 = Fatigue, PHQ5 = Appetite changes, PHQ6 = Feeling of worthlessness, PHQ7 = Concentration difficulties, PHQ8 = Psychomotor agitation/retardation, PHQ9 = Thoughts of death, PSU1 = Missing planned work, PSU2 = Hard concentrating, PSU3 = Feeling pain, PSU4 = Can’t stand not having a phone, PSU5 = Impatient without phone, PSU6 = Having phone in mind, PSU7 = Never give up using a phone, PSU8 = Afraid to miss conversations, PSU9 = Longer than intended, PSU10 = Use the phone too much. The first column contains variables in the network at T1, and the first row contains variables in the network at T2.

| **Time 1** | **Time 2** |
| --- | --- |
| **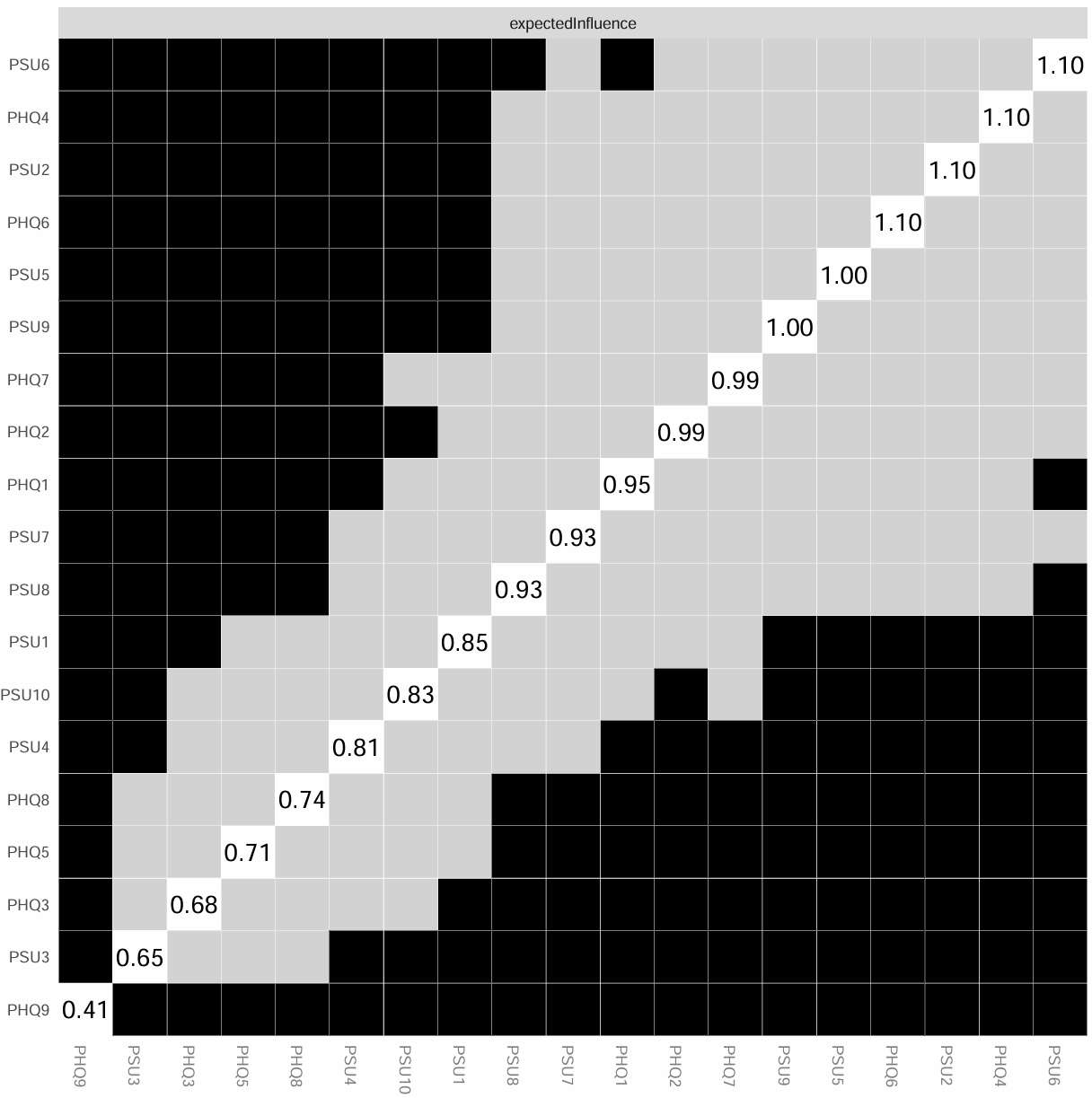** | **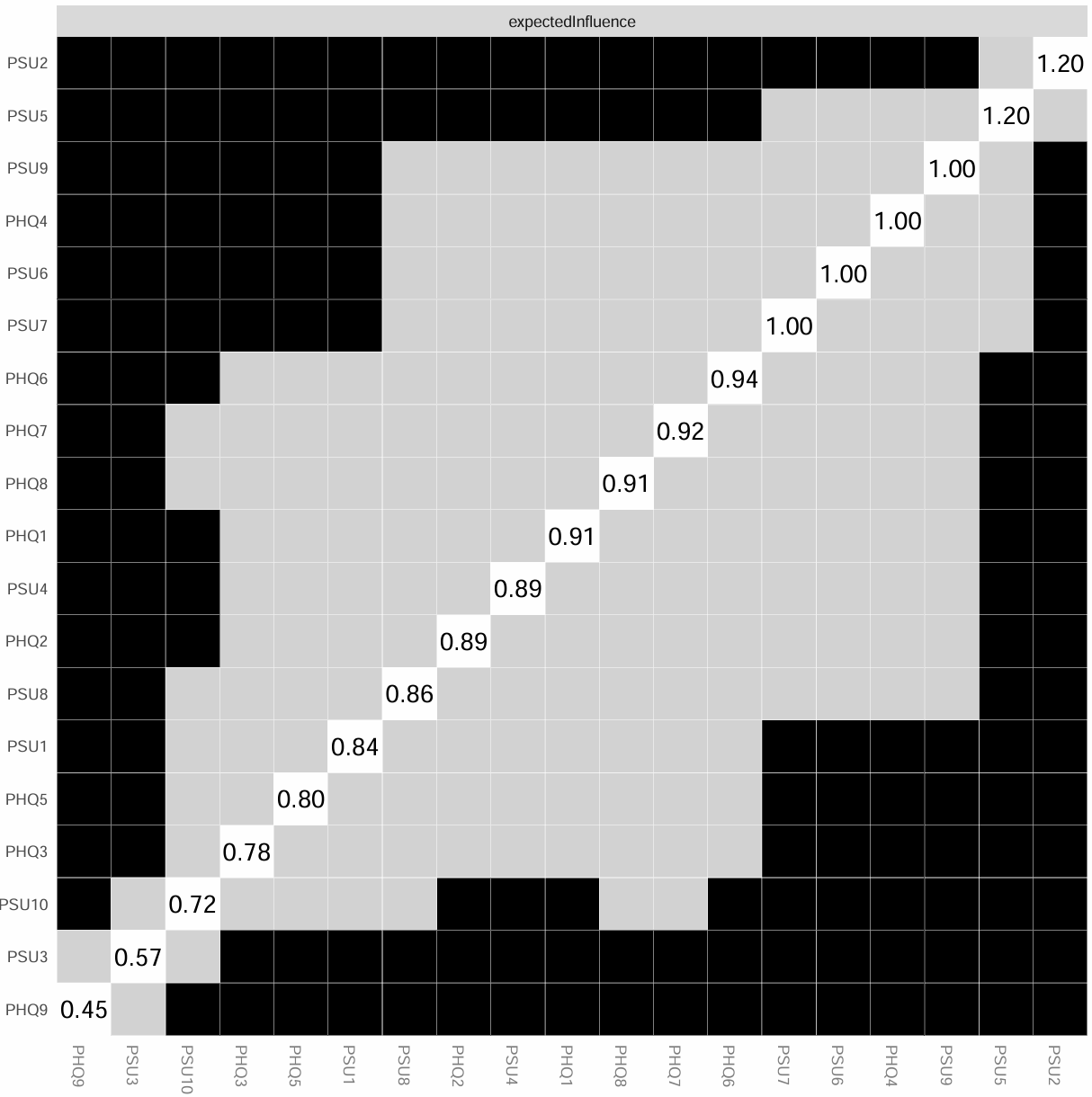** |

Figure S1. Bootstrapped difference tests for the node’s expected influence of the contemporaneous networks across Time 1 to Time 2.

*Note.* PHQ1 = Anhedonia, PHQ2 = Depressed or sad mood, PHQ3 = Sleep difficulties, PHQ4 = Fatigue, PHQ5 = Appetite changes, PHQ6 = Feeling of worthlessness, PHQ7 = Concentration difficulties, PHQ8 = Psychomotor agitation/retardation, PHQ9 = Thoughts of death, PSU1 = Missing planned work, PSU2 = Hard concentrating, PSU3 = Feeling pain, PSU4 = Can’t stand not having a phone, PSU5 = Impatient without phone, PSU6 = Having phone in mind, PSU7 = Never give up using a phone, PSU8 = Afraid to miss conversations, PSU9 = Longer than intended, PSU10 = Use the phone too much. Grey boxes indicate no significant difference, whereas black boxes indicate a statistically significant difference (*p* < 0.05).

| **Time 1** | **Time 2** |
| --- | --- |
| **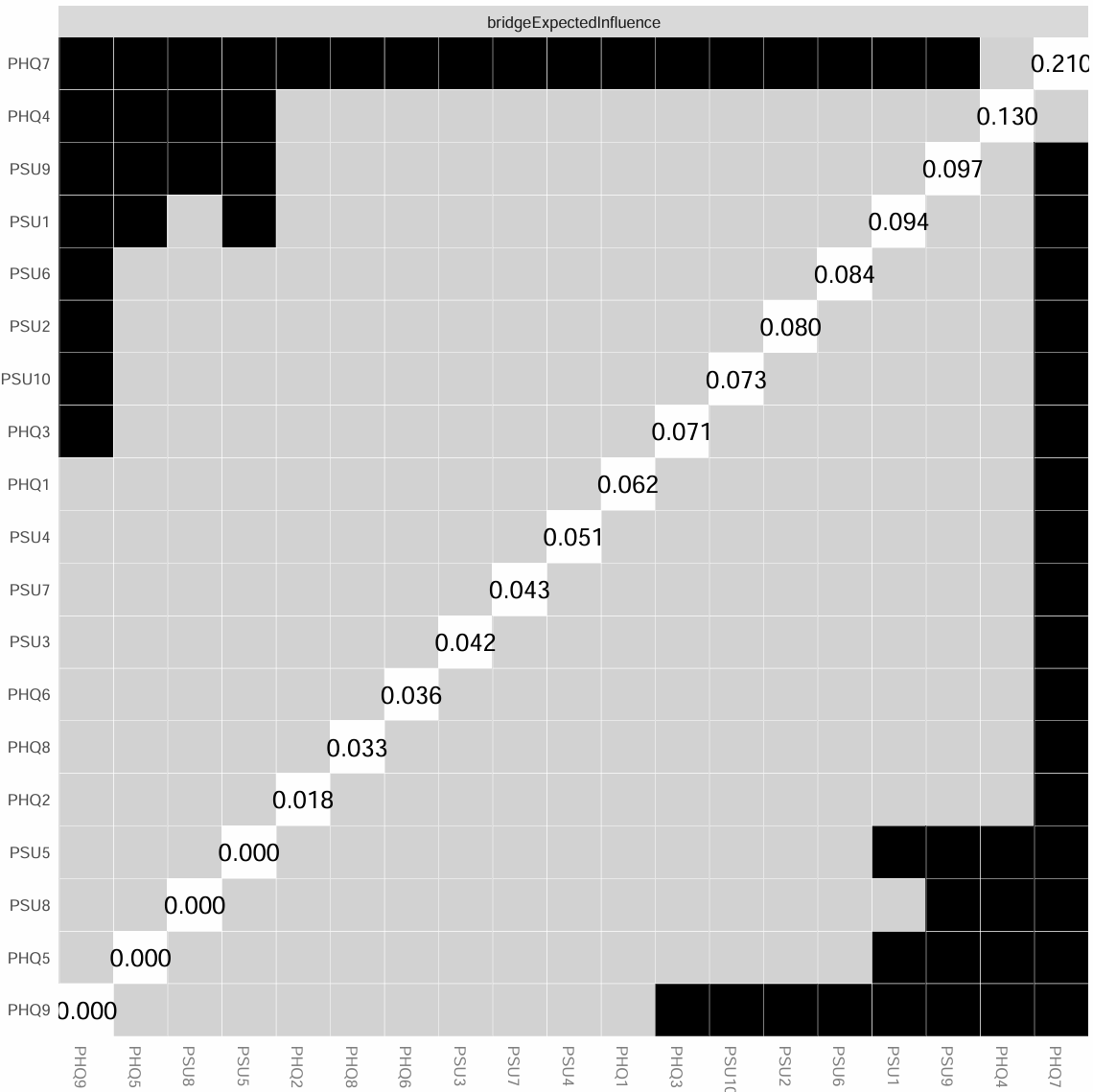** | **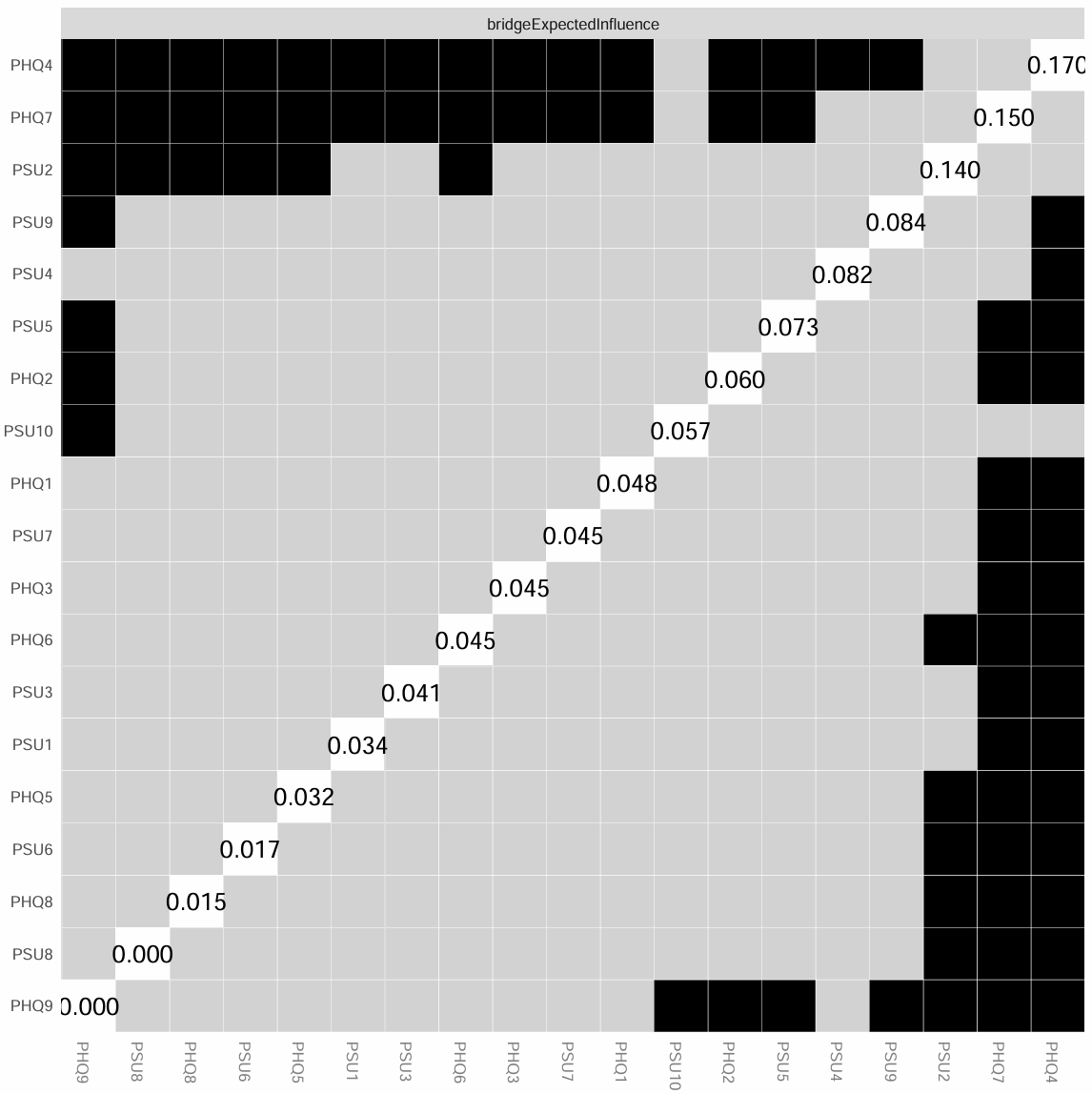** |

Figure S2. Bootstrapped difference tests for the node’s bridge expected influence of the contemporaneous networks across Time 1 to Time 2.

*Note*. PHQ1 = Anhedonia, PHQ2 = Depressed or sad mood, PHQ3 = Sleep difficulties, PHQ4 = Fatigue, PHQ5 = Appetite changes, PHQ6 = Feeling of worthlessness, PHQ7 = Concentration difficulties, PHQ8 = Psychomotor agitation/retardation, PHQ9 = Thoughts of death, PSU1 = Missing planned work, PSU2 = Hard concentrating, PSU3 = Feeling pain, PSU4 = Can’t stand not having a phone, PSU5 = Impatient without phone, PSU6 = Having phone in mind, PSU7 = Never give up using a phone, PSU8 = Afraid to miss conversations, PSU9 = Longer than intended, PSU10 = Use the phone too much. Grey boxes indicate no significant difference, whereas black boxes indicate a statistically significant difference (*p* < 0.05).

| **Time 1** | **Time 2** |
| --- | --- |
| **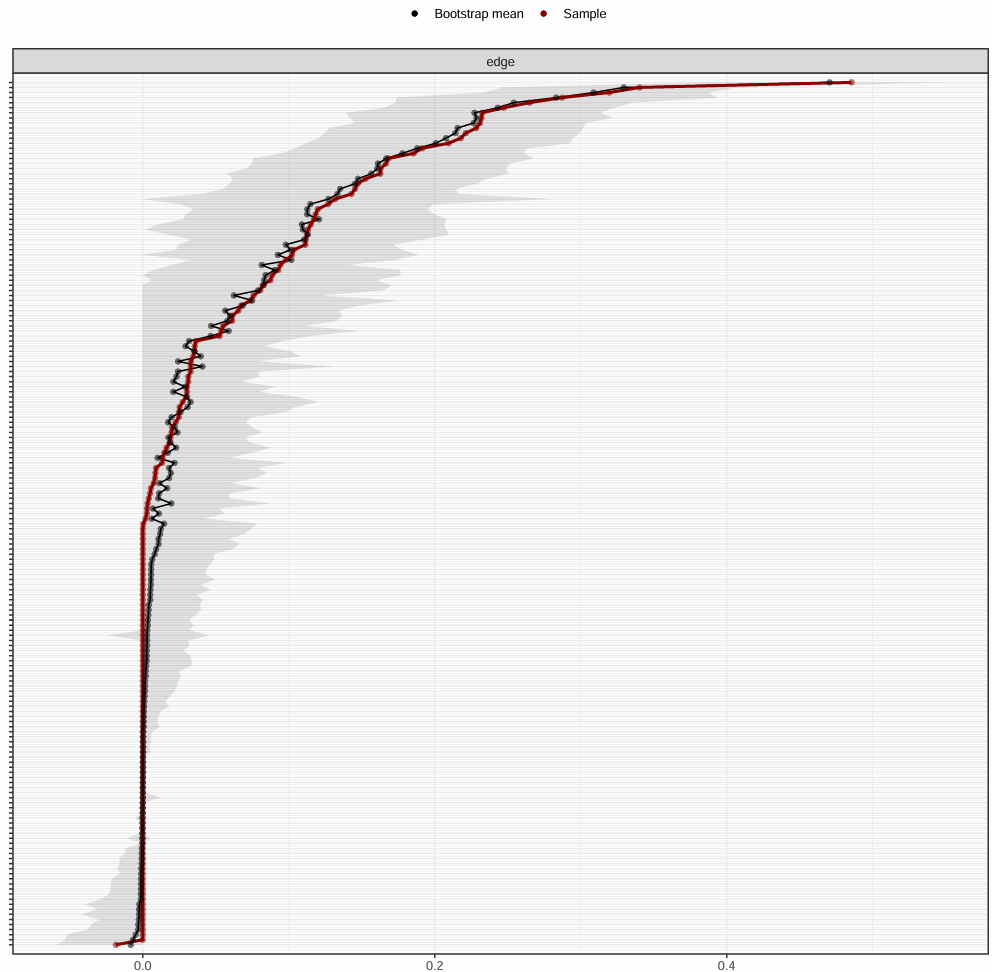** | **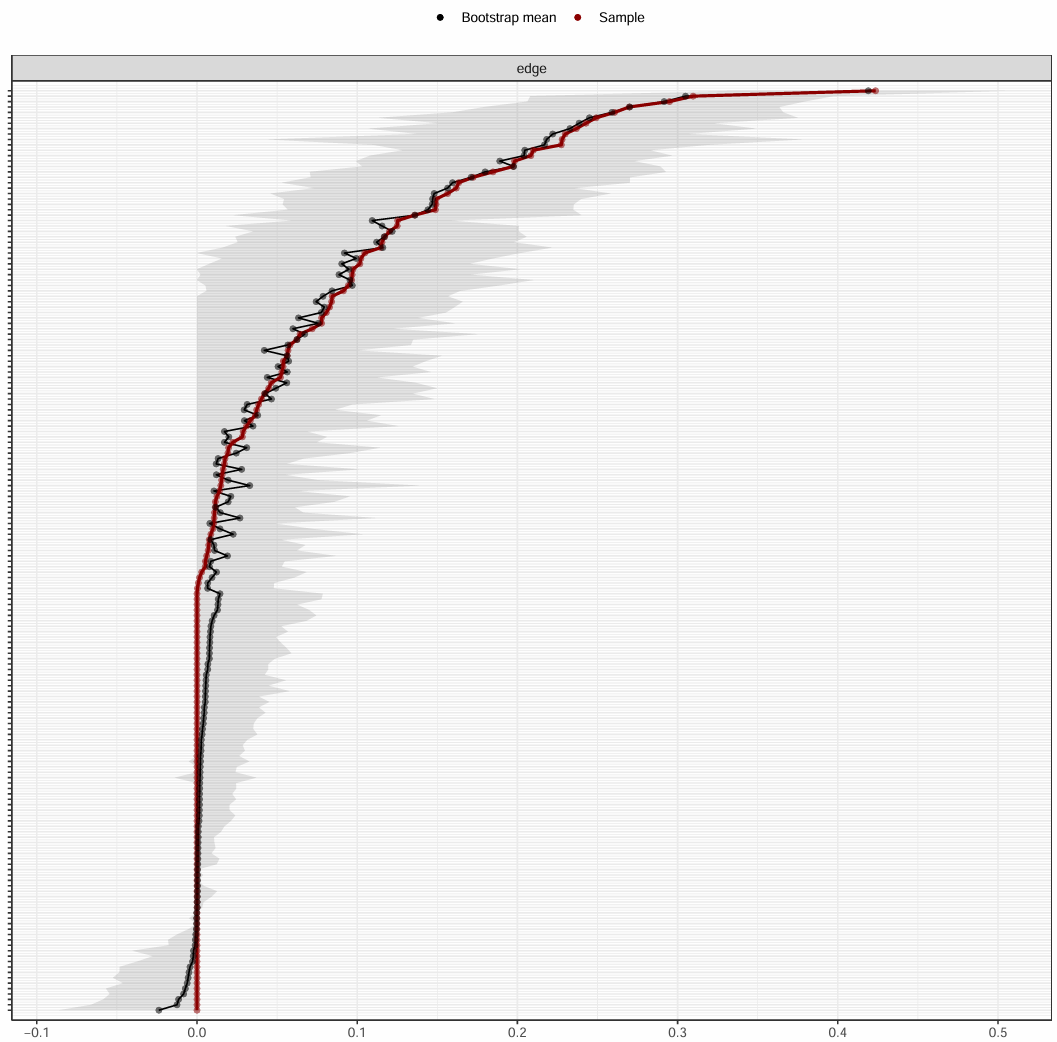** |

Figure S3*.* Accuracy of the edge estimates of the contemporaneous networks across Time 1 to Time 2.

Each horizontal line represents one edge of the network. The red point and black point on each horizontal line represent the sample values of the edge and the bootstrap mean of the edge, respectively. The gray line on each horizontal line represents the bootstrapped CIs of the edge, and narrower gray line means better accuracy of the edge.

| **Time 1** | **Time 2** |
| --- | --- |
| **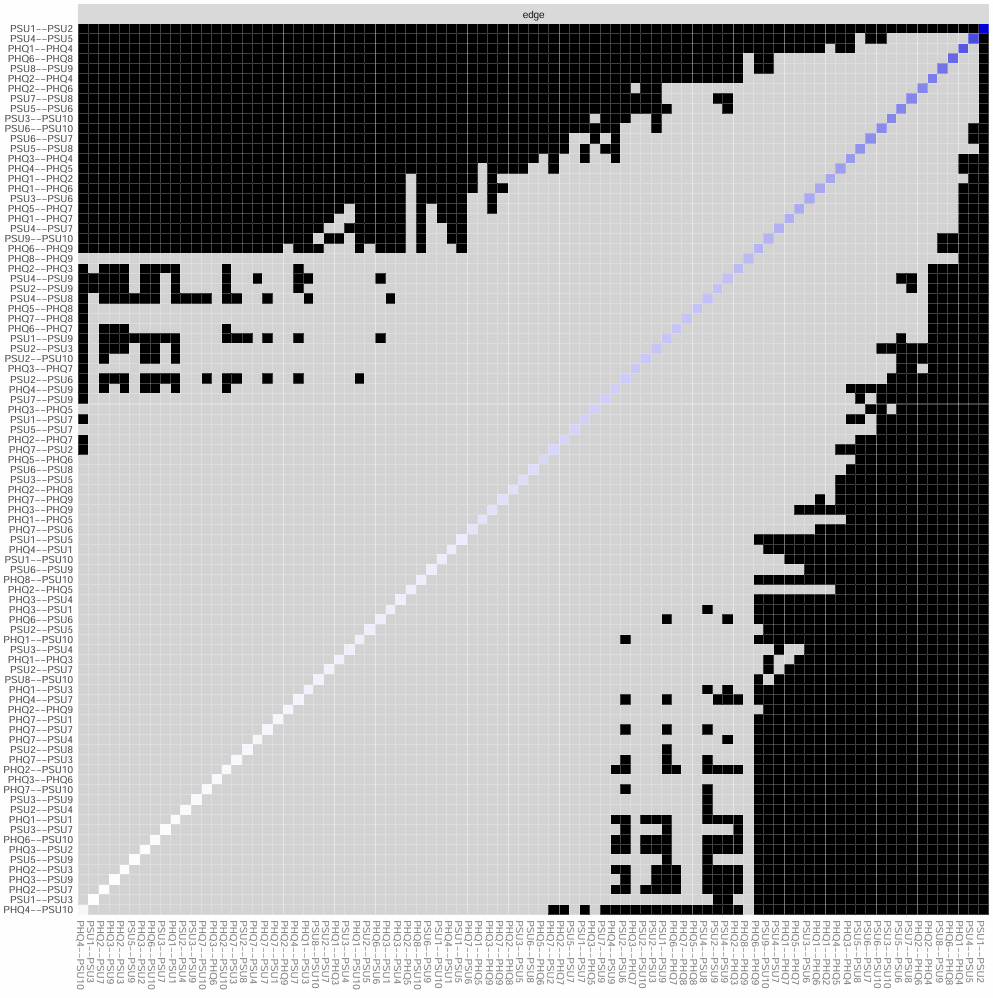** | **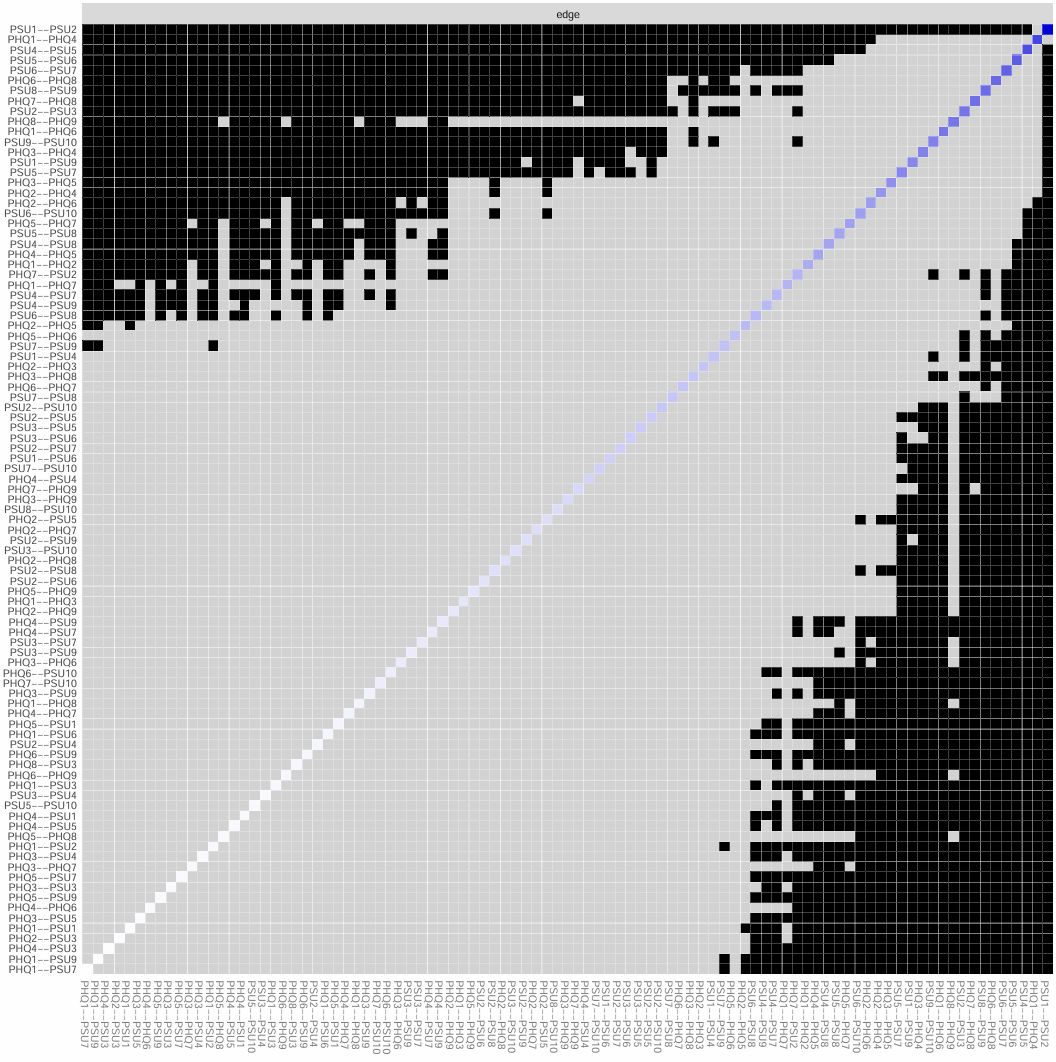** |

Figure S4. Bootstrapped difference tests (α = 0.05) between edges of the contemporaneous networks across Time 1 to Time 2

*Note*. PHQ1 = Anhedonia, PHQ2 = Depressed or sad mood, PHQ3 = Sleep difficulties, PHQ4 = Fatigue, PHQ5 = Appetite changes, PHQ6 = Feeling of worthlessness, PHQ7 = Concentration difficulties, PHQ8 = Psychomotor agitation/retardation, PHQ9 = Thoughts of death, PSU1 = Missing planned work, PSU2 = Hard concentrating, PSU3 = Feeling pain, PSU4 = Can’t stand not having a phone, PSU5 = Impatient without phone, PSU6 = Having phone in mind, PSU7 = Never give up using a phone, PSU8 = Afraid to miss conversations, PSU9 = Longer than intended, PSU10 = Use the phone too much. Gray boxes indicate the edge do not differ significantly from one-another, and black boxes represent the edge differ significantly from one-another.

| **Time 1** | **Time 1** |
| --- | --- |
| **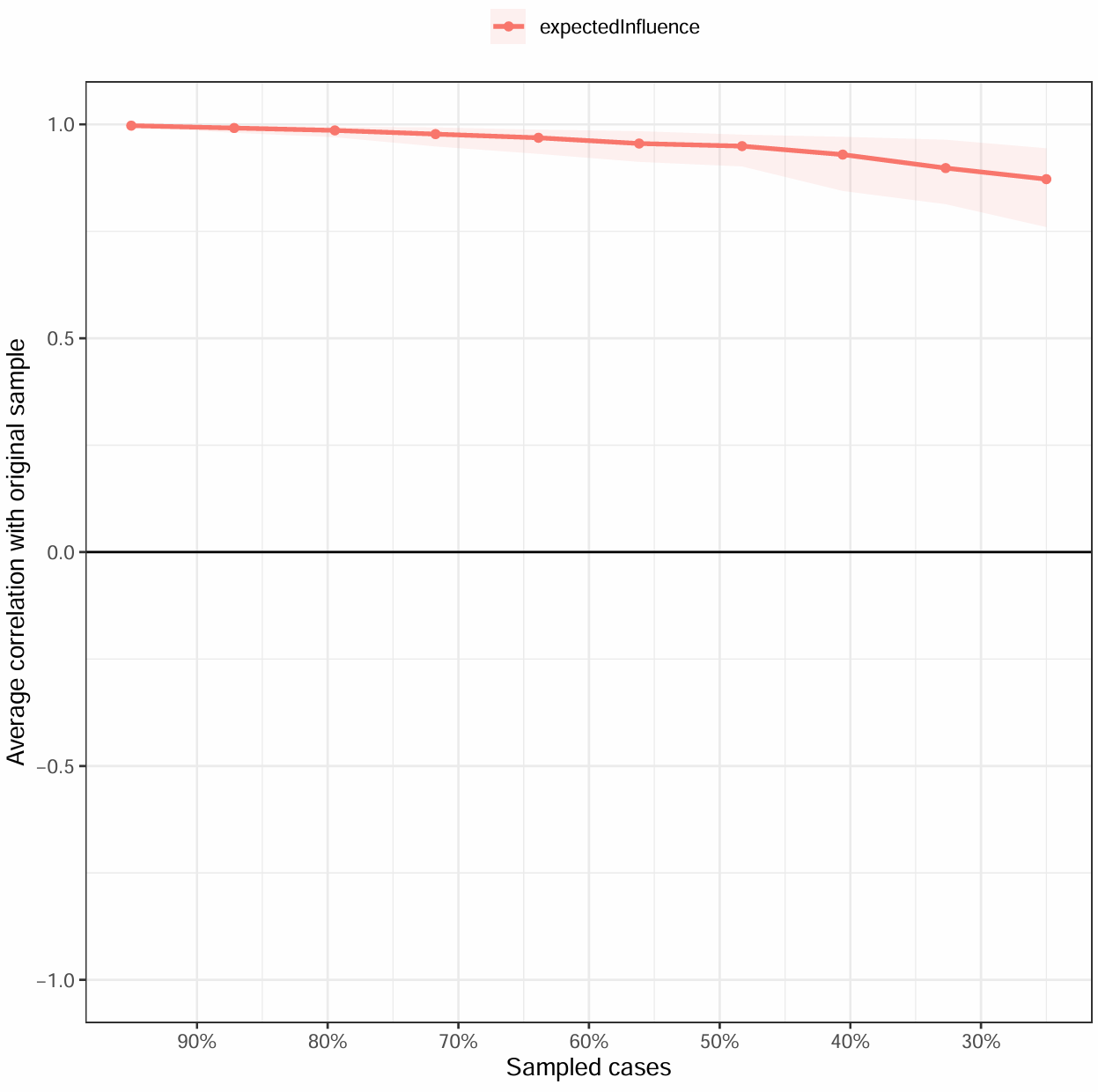** | **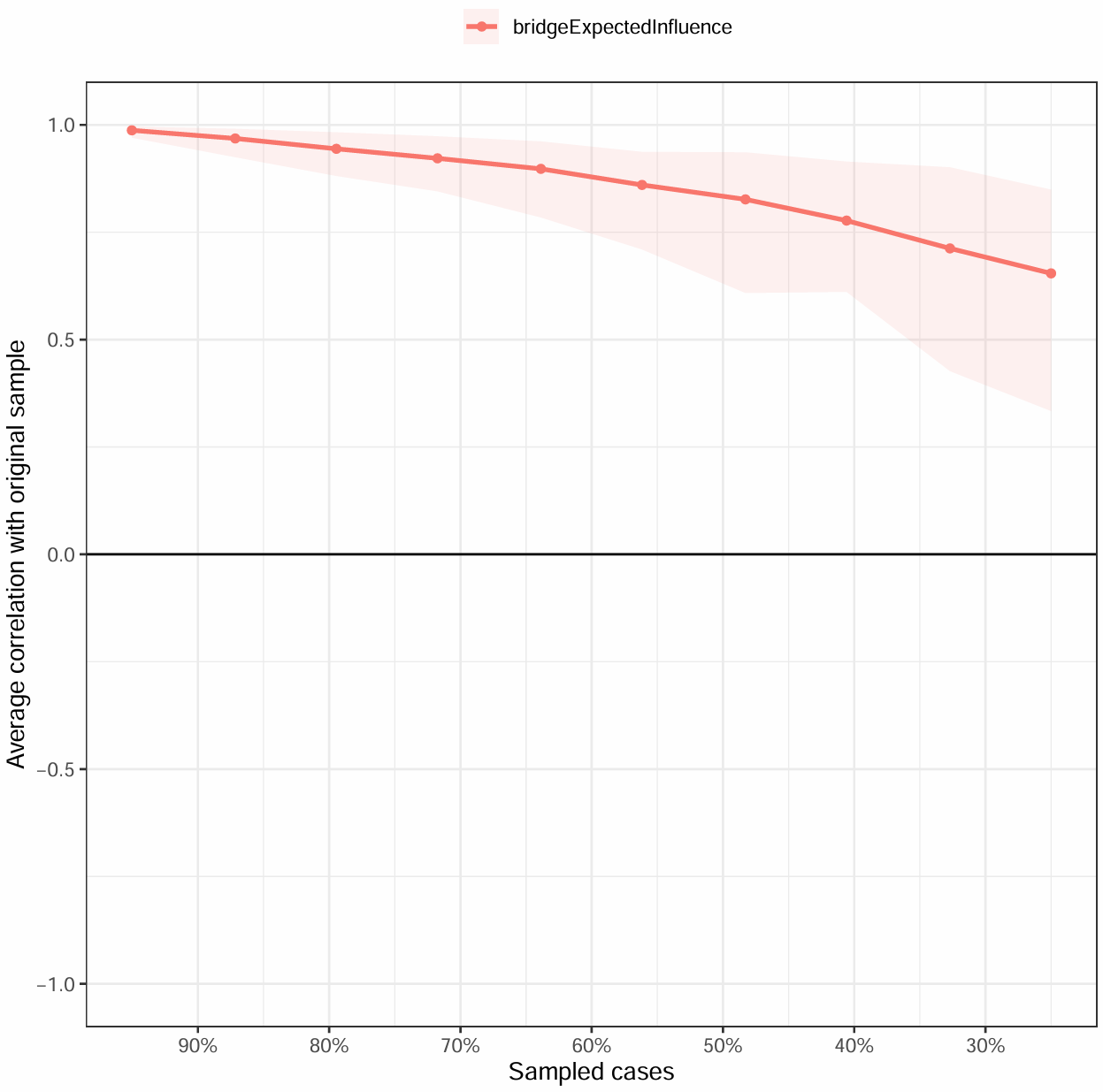** |
| **Time 2** | **Time 2** |
| **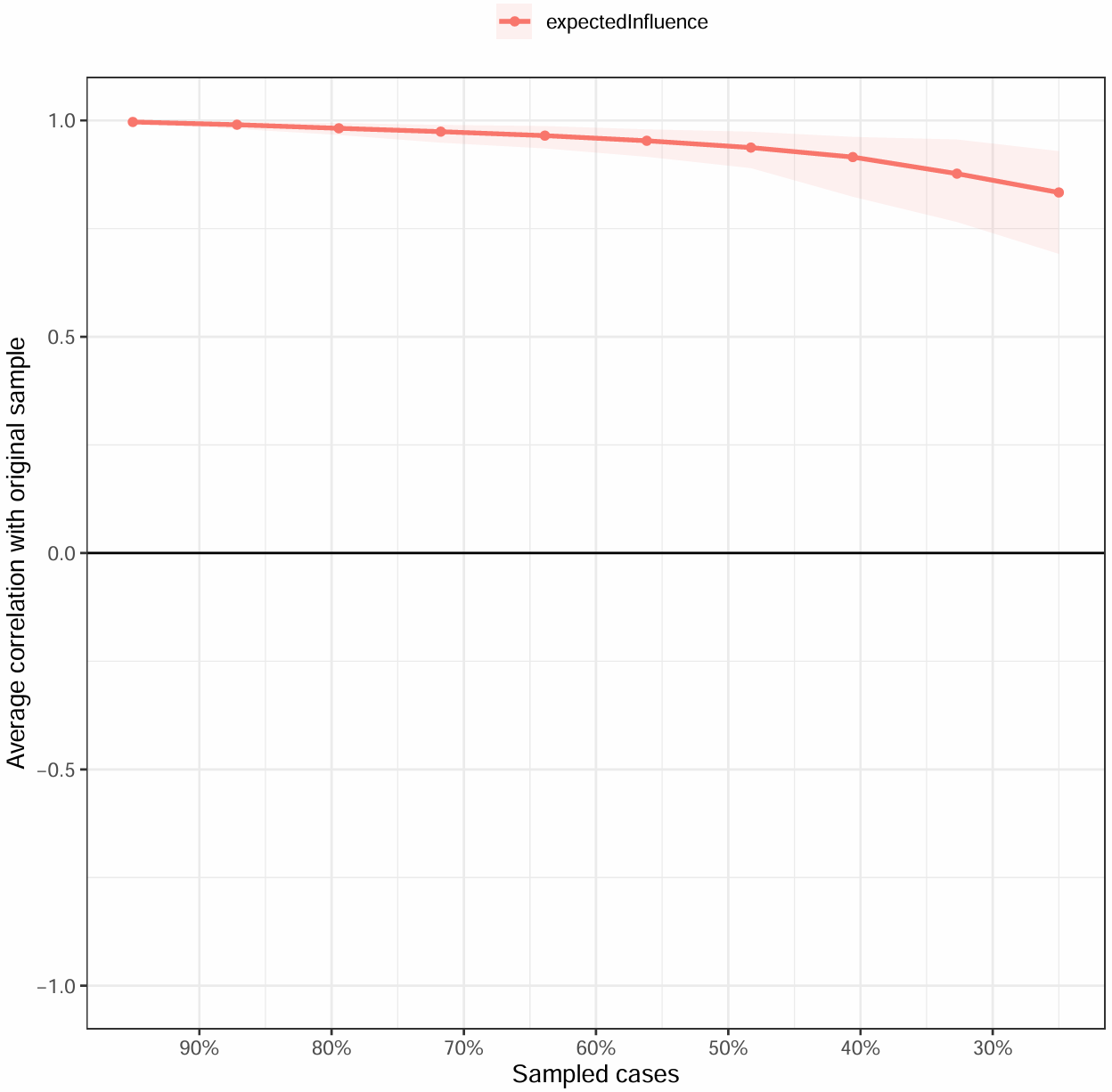** | **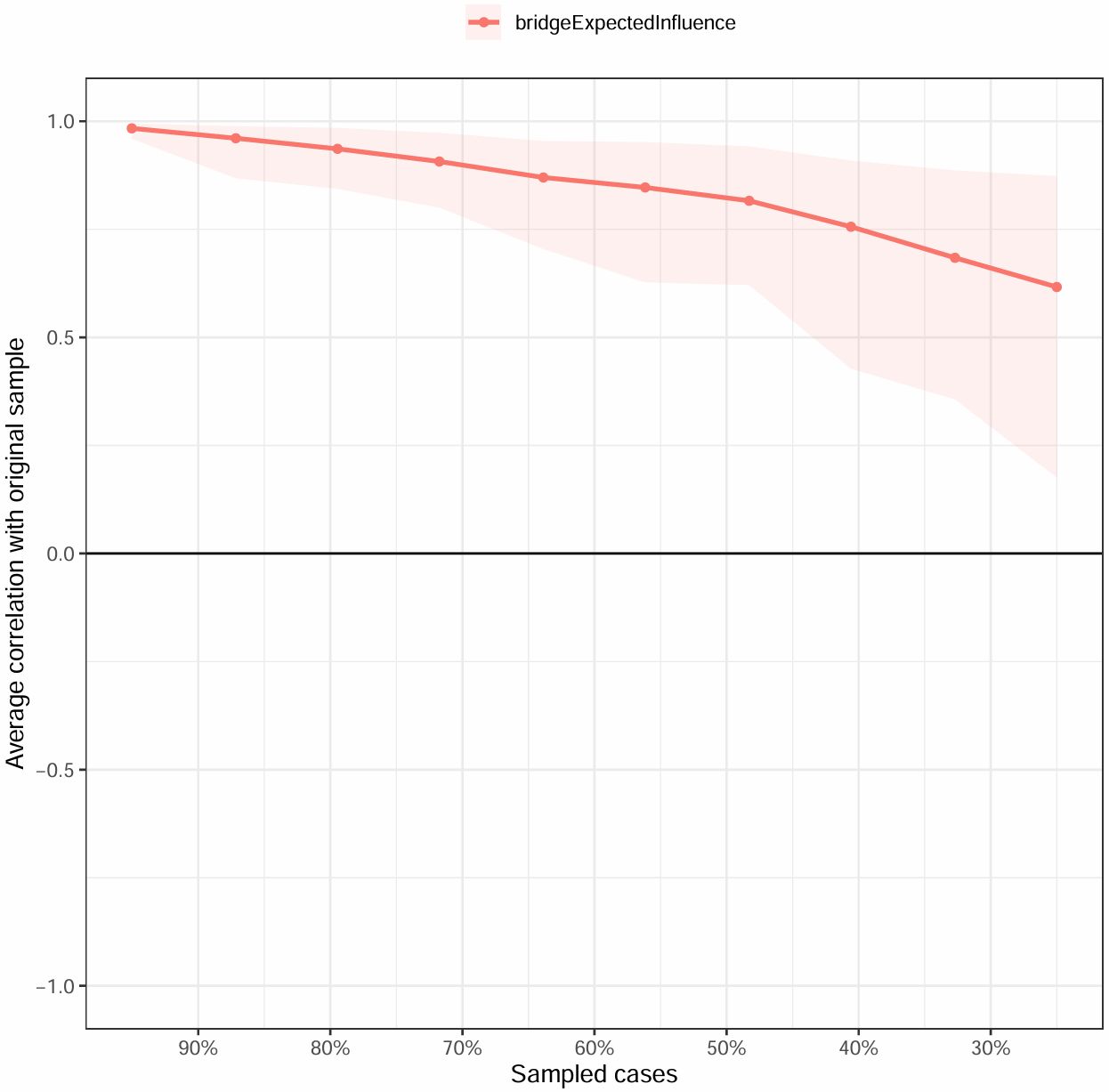** |

Figure S5. Stability of central indices of the contemporaneous networks across Time 1 to Time 2. The x-axis represents the percent of dropped case on the original sample. The y-axis represents the correlation between rest sample and original sample. Lines indicate the means and areas indicate the range from the 2.5th quantile to the 97.5th quantile.

| Out-expected influence | In-expected influence |
| --- | --- |
| **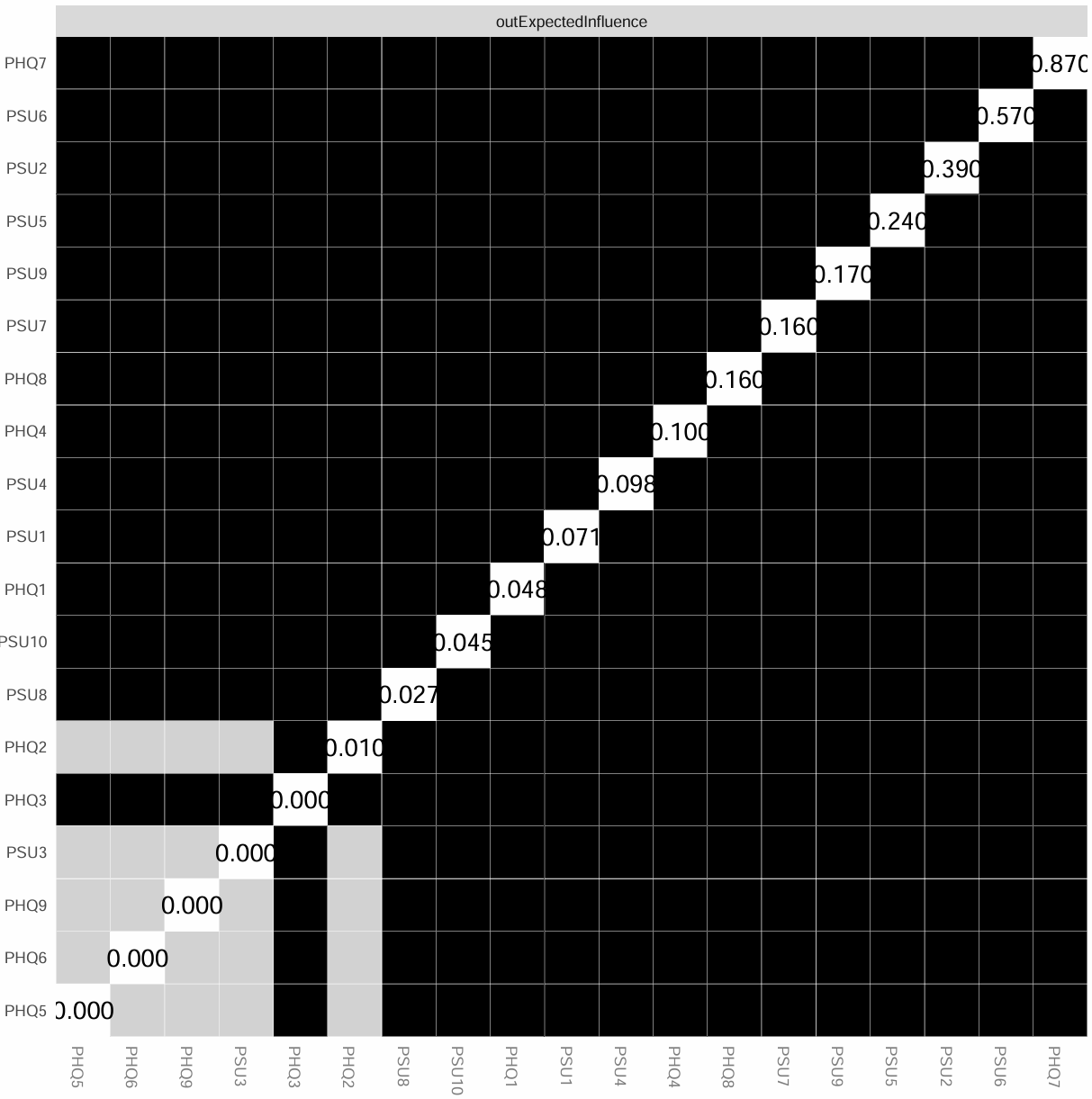** | **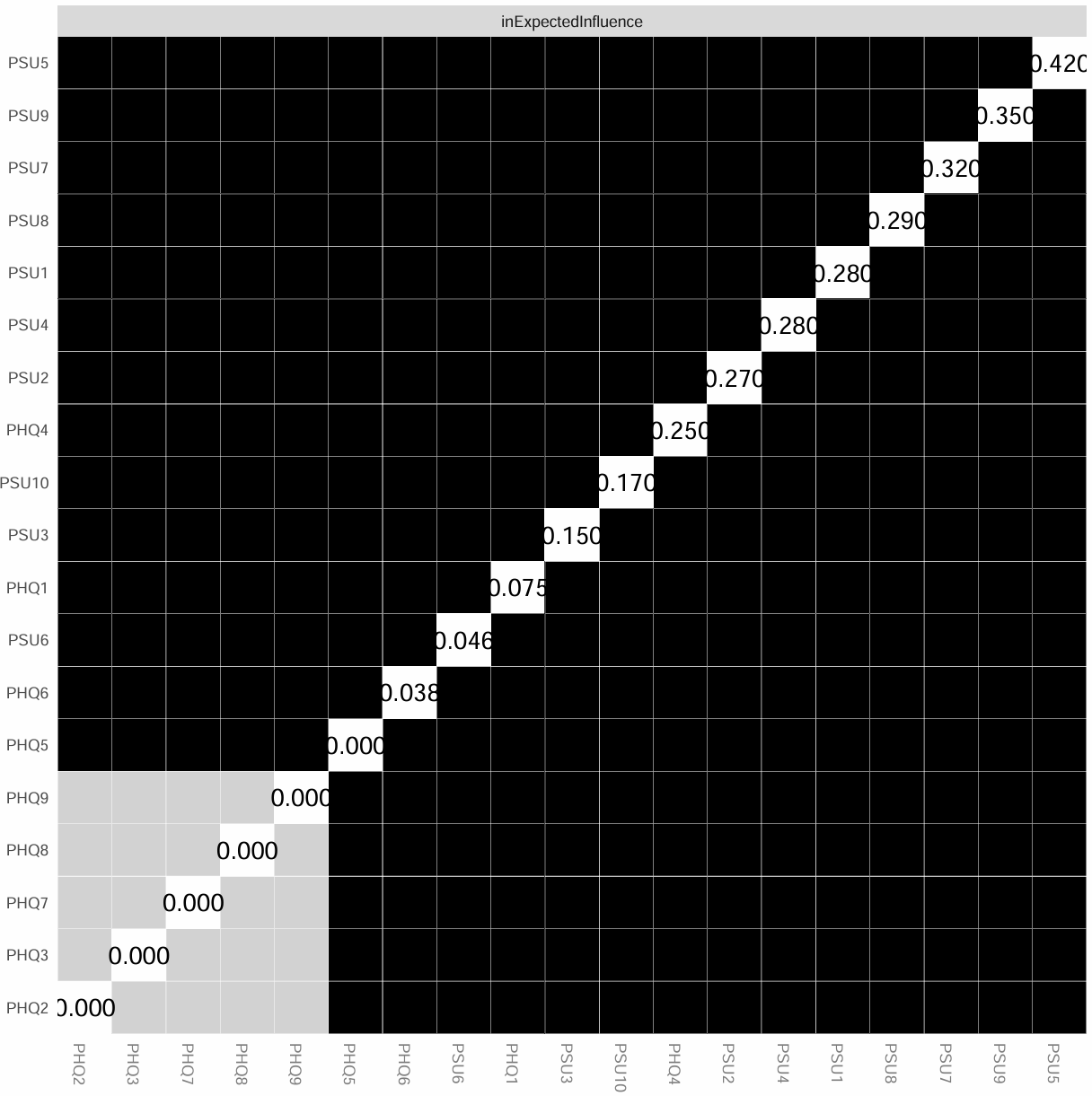** |
| Bridge expected influence | |
| **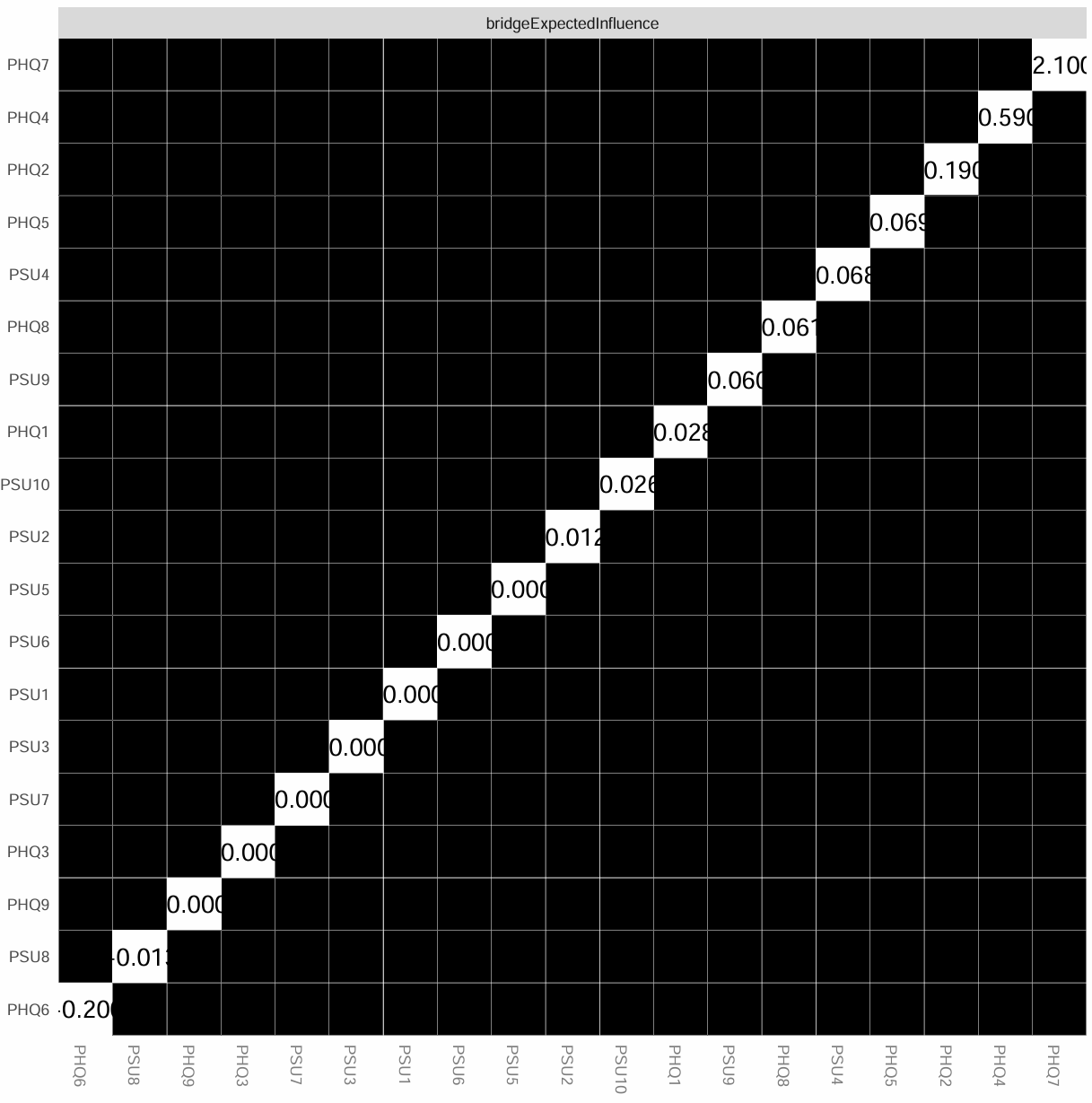** | |

Figure S6. Bootstrapped difference tests for the node’s out-expected influence and in-expected influence of the cross-lagged panel network. Note. PHQ1 = Anhedonia, PHQ2 = Depressed or sad mood, PHQ3 = Sleep difficulties, PHQ4 = Fatigue, PHQ5 = Appetite changes, PHQ6 = Feeling of worthlessness, PHQ7 = Concentration difficulties, PHQ8 = Psychomotor agitation/retardation, PHQ9 = Thoughts of death, PSU1 = Missing planned work, PSU2 = Hard concentrating, PSU3 = Feeling pain, PSU4 = Can’t stand not having a phone, PSU5 = Impatient without phone, PSU6 = Having phone in mind, PSU7 = Never give up using a phone, PSU8 = Afraid to miss conversations, PSU9 = Longer than intended, PSU10 = Use the phone too much. Black boxes indicate nodes that significantly differ in centrality (p < .05), and gray boxes indicate nodes whose centrality does not significantly differ.


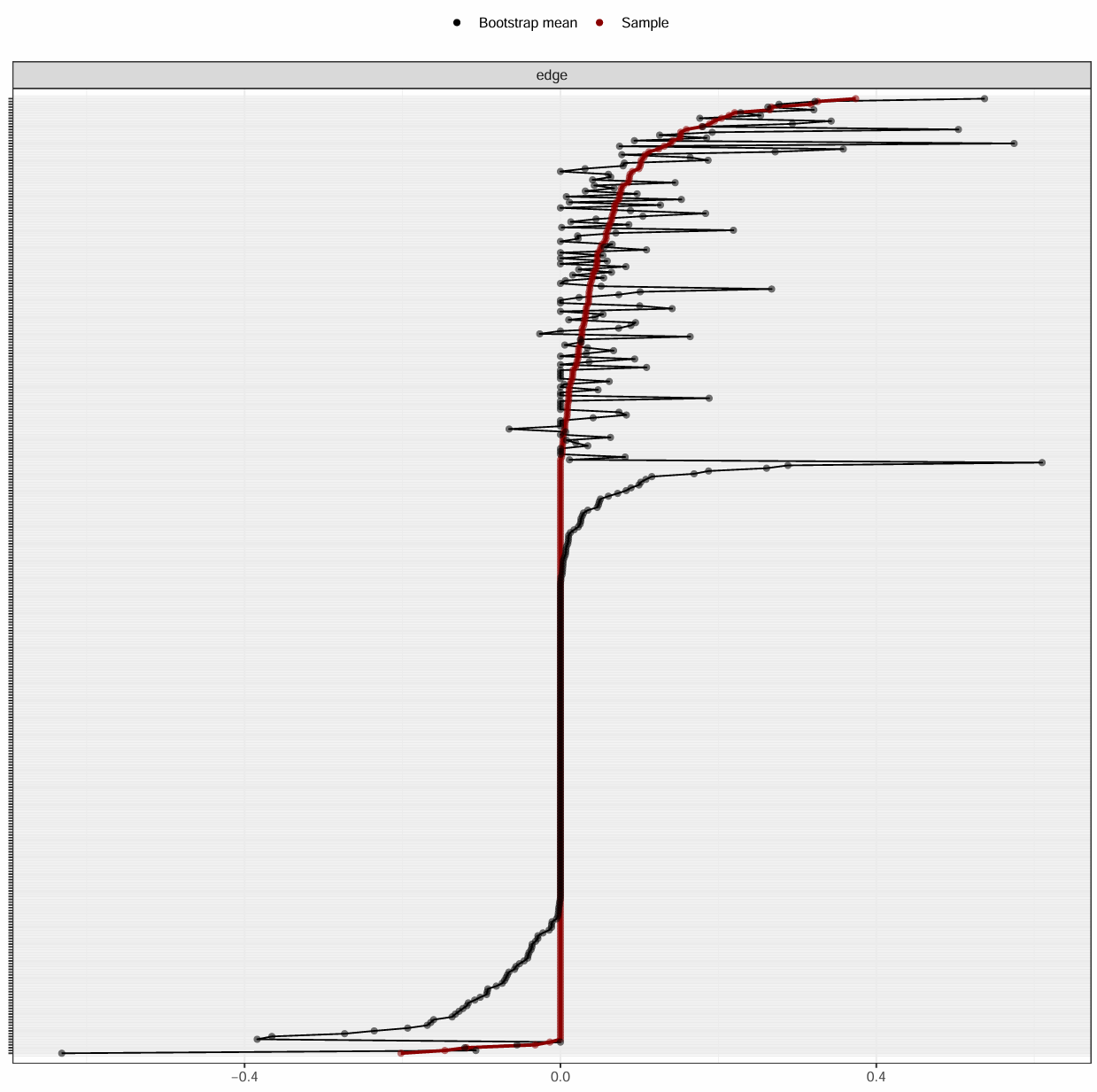


Figure S7. Accuracy of the edge estimates of the cross-lagged panel network. Each horizontal line represents one edge of the network. The red point and gray point on each horizontal line represent the sample values of the edge and the bootstrap mean of the edge, respectively. The gray line on each horizontal line represents the bootstrapped CIs of the edge, and narrower gray line means better accuracy of the edge.


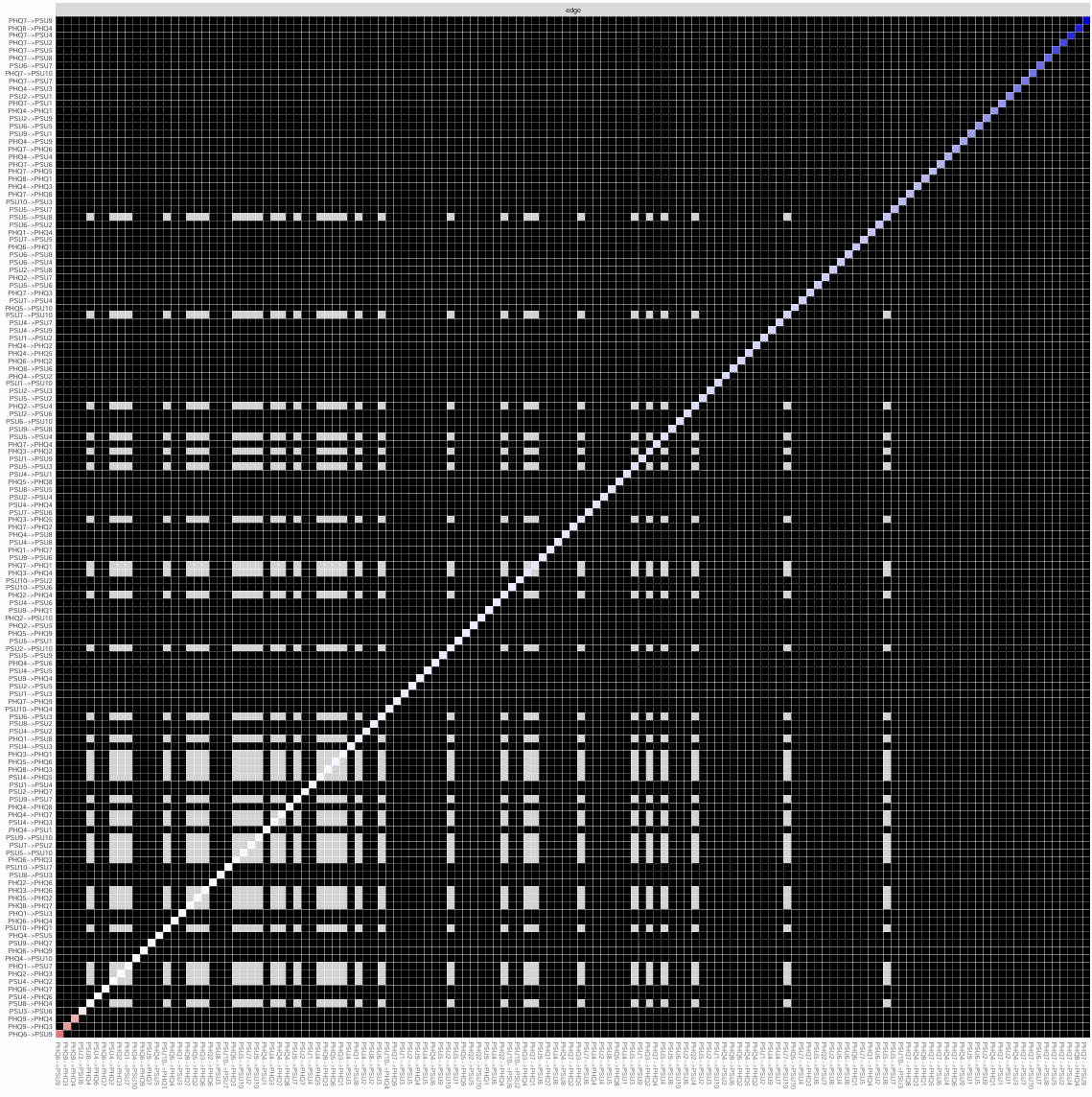


Figure S8. Bootstrapped difference tests (α = 0.05) between edges of the cross-lagged panel network. Black boxes indicate edges that significantly differ from each other (p < .05), and gray boxes indicate edges that do not significantly differ.


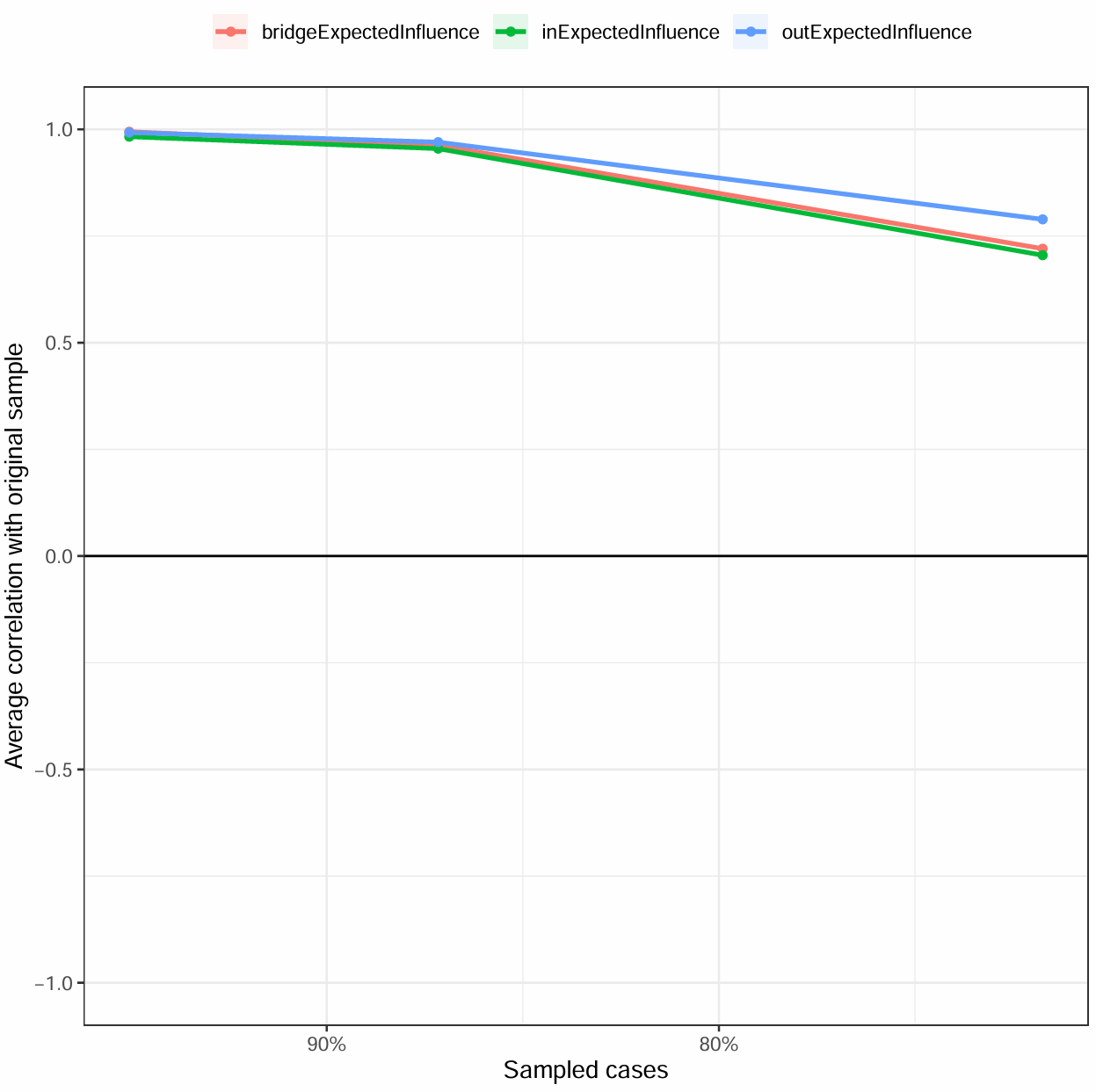


Figure S9. Stability of central indices of the cross-lagged panel network. The x-axis represents the percent of dropped case on the original sample. The y-axis represents the correlation between rest sample and original sample. Lines indicate the means and areas indicate the range from the 2.5th quantile to the 97.5th quantile.
